# Rare disease education in medical schools: patient-centered and innovative strategies

**DOI:** 10.1186/s13023-025-03771-8

**Published:** 2025-11-20

**Authors:** Sharon Huynh, Eric L. Wan, Angelette Pham, Robin Yoon, Scott Dorris, Nada Yazigi, Jessica M. Jones

**Affiliations:** 1 Georgetown University School of Medicine, 3900 Reservoir Rd NW, Washington, DC 20007 USA; 2 College of Arts and Sciences, Georgetown University, Washington, DC USA; 3 MedStar Georgetown Transplant Institute, Washington, DC USA; 4 Department of Biochemistry and Molecular Biology and Cellular Biology, Georgetown University School of Medicine, Washington, DC USA

**Keywords:** Rare Diseases, Medical Education, Patient Panel, Scoping Review, Undergraduate Medical Education

## Abstract

**Purpose:**

Globally, approximately 300 million people live with a rare disease, while in the United States, nearly 30 million, or 1 in 10 Americans, have a rare disease or disorder (RD) (The Lancet Global Health. Lancet Glob Health 2024. 10.1016/S2214-109X(24)00244-0; National Center for Advancing Translational Sciences. Rare disease day at NIH 2024; 2024). Despite the prevalence of RDs, many physicians do not have adequate awareness of RDs or training to care for RD patients. RD advocates are focusing on undergraduate medical education to improve timely and quality care for RD patients. Thus, in 2023, the authors conducted an RD patient panel at their medical school and a scoping review of global medical schools’ approaches to integrating RD education into their curricula. While the panel was implemented in a U.S. context, the scoping review revealed diverse approaches applicable worldwide, highlighting the need to prepare medical students for the global burden of RDs, which affect approximately 300 million people across WHO regions, with significant challenges in low- and middle-income countries (LMICs) (The Lancet Global Health. Lancet Glob Health 2024. 10.1016/S2214-109X(24)00244-0).

**Method:**

In 2023, the authors implemented an RD patient and caregiver panel for first-year medical students (*n* = 175) and Special Master’s Program students (*n* =109). Pre- and post-panel surveys with ANOVA statistical analysis assessed attitudes toward and knowledge of RDs. The systematic scoping review from the databases MEDLINE, Embase, ERIC, and Web of Science sought international literature written in English discussing RD education in medical schools. From 11 relevant studies, the authors analyzed broad trends in RD education and their outcomes.

**Results:**

Pre- and post-survey results from the patient panel demonstrated improved students’ attitude, knowledge, and value of RDs. Confidence in caring for RD patients also increased significantly. From the scoping review, educational intervention types and content varied broadly among medical schools. Many institutions employed multiple interventions simultaneously, and all reported positive attitudes, higher knowledge from participants, or both. However, interventions lacked input from RD patients and caregivers.

**Conclusions:**

Patient panels improved students’ understanding of the RD patient experience. Beyond patient panels, effective approaches in RD education in undergraduate medical curricula included lectures, case studies, role-playing simulations, artificial intelligence, and more. The authors hope medical schools will continue to implement RD education into their curricula.

**Supplementary Information:**

The online version contains supplementary material available at 10.1186/s13023-025-03771-8.

## Introduction

The world needs to know that we have a voice... to be understood and to be taken seriously in the medical community and by others around us.

- Patient diagnosed with Abetalipedemia, a rare metabolic disorder

Rare diseases (RDs), while individually rare, collectively affect nearly 300 individuals worldwide and approximately 30 million Americans, highlighting the urgent need for heightened awareness and understanding [1]. As underscored by a patient with abetalipoproteinemia, increased education among medical students and healthcare professionals is paramount. This education demonstrates the importance of early diagnosis and proper treatment of RDs and holds the potential for groundbreaking advancements in therapeutic approaches. Our manuscript aims to bridge the gap in RD care by amplifying patient voices and emphasizing the transformative impact of comprehensive education initiatives within the medical community. Through this advocacy, we strive to foster inclusivity, empathy, and effective healthcare practices, ensuring support for all individuals, regardless of the rarity of their condition.

The Genetic and Rare Diseases Information Center defines an RD as a disease or condition that impacts fewer than 200,000 people in the U.S [2]. Conservative estimates place the global prevalence of RDs as high as 446 million affected, excluding cancers, infectious diseases, and poisonings [3]. In the U.S., RDs affect approximately 1 in 10 Americans, and the NIH considers them a “public health crisis.” [1] While there are 10,000 individual RDs, according to the NIH, it is a misconception that physicians and medical trainees will not encounter patients with RDs at some point in their careers [1].

Physicians and trainees may be intimidated by RDs. However, we wish to remind readers that RD patients face significant challenges during the diagnostic journey and generally welcome physician allyship. The average RD patient waits six years before receiving a definitive and accurate diagnosis [4]. During their diagnostic journey, patients can expect to have 17 doctor visits on average between symptom onset and diagnosis [5].

The challenging diagnostic journey has a significant economic burden for RD patients, families, and society. On an individual patient level, delayed diagnosis results in up to $517,000 in avoidable costs and a significant barrier to work, with patients missing up to 231 days of work on average, depending on the RD, due to clinical visits [5]. From a societal perspective, the total economic burden of a subset of just 379 RDs is at least $997 billion, driven by costs associated with hospital inpatient stays, prescription medication, and labor market productivity losses [5].

Lack of RD education, referral protocols for RD patients, and knowledge about where to refer them are potential contributing factors to the delayed diagnosis. Unfortunately, primary care physicians were twice as likely as specialists to be reluctant to involve themselves in diagnosing RD [6]. Only 19% of primary care physicians self-reported their knowledge of RDs as good or excellent at the time of diagnosis, compared to 59% of specialists [6]. 57% of primary care physicians rated their training in RD as neutral, ineffective, or very ineffective compared to 40% of specialists [6].

Globally, RDs impact an estimated 300 million individuals, with over 7000 distinct conditions, 80% of which are genetic and 70% presenting in childhood; in LMICs, where healthcare resources are scarce, the burden is exacerbated by limited diagnostic tools and treatments, with 95% of RDs lacking approved therapies and a diagnostic delay averaging 4.8 years. These gaps highlight the need for structural solutions beyond individual physician training, such as establishing multi-center registries for RDs and specialized clinics dedicated to referral and management of suspected cases, which could pool data and expertise to facilitate early detection, improve care, and enhance medical education [7].

The impact of insufficient training might be most profoundly felt during the clinical encounter that intends to forge strong patient-physician relationships. The Association of American Medical Colleges (AAMC)’s recommendations for clinical practice competency include “the ability to… build a physician–patient relationship for the purposes of information gathering, guidance, education and support. This competency… necessarily includes the ability also to build relationships with peers, teachers, healthcare professionals, and others who may be involved in the care of patients and in the education thereof” [8]. However, for RD patients, cultivating such relationships has proven challenging.

In 2022, a groundbreaking healthcare access survey administered to the RD patient community revealed that 46% of physicians were unwilling to ask local or regional physicians for help in making a diagnosis [9]. Thus, RD patients must often become self-reliant, conduct their own research, find their own care and advocacy networks, and bring forth suspected conditions to their physicians. Patients often become experts on their conditions and educate their care teams, so patient-directed interaction is common for RD patients [10]. However, contrary to expectations, 47% of physicians were unwilling to consider RD patients’ suggestions, and 37% of patients and caregivers rated their physician as unwilling to investigate the causes of their symptoms [9]. Can we truly expect that RD patients will find the strong patient-physician relationships that they, like all patients, deserve? Not surprisingly, 45% of patients and caregivers rated their physicians’ knowledge of RD as “poor” [9]. Clearly, there is room for improvement.

The National Organization for Rare Disorders (NORD) initiated student advocacy programs to provide early RD exposure for the next generation of healthcare professionals. Many medical schools also recognize that exposure to RD begins during undergraduate medical education and provide early education on RD patients. NORD hopes to establish student advocacy programs and curricular reform at medical schools associated with each of their 20–25 Centers of Excellence by the beginning of 2025. However, the most effective way to provide this exposure to trainees is unclear.

We, therefore, sought to understand the landscape of RD medical education through a systematic scoping review to identify RD medical education interventions and their potential impact on students, patients, or families. We also sought to understand the impact of an inaugural RD patient panel at Georgetown University School of Medicine (GUSOM)’s first block of first-year preclinical medical education. We integrated our findings into practical recommendations for medical schools and other stakeholders. We aimed to encourage those in medical education to pursue practical avenues of increasing RD exposure to trainees. We hoped to collectively change the narrative around clinical treatment of RDs and expedite diagnosis for RD patients.

## Methods

### Panel Event Evaluation

We implemented a 2-h RD panel event into the Medical Biochemistry curriculum at GUSOM, a course taken by first-year medical students (M1, *n* =175) alongside Special Master’s Program (SMP) students (*n* =109); SMPs students are graduate students enhancing their credentials for medical school or health profession applications, with 85% of SMP students matriculating into medical school post-program. We intentionally selected first-year students during their initial preclinical block to establish a baseline understanding of their attitudes and knowledge about RDs early in their medical education. This approach allows us to capture their perspectives before significant exposure to clinical training, providing insight into foundational gaps that curriculum designers can address to better prepare students for RD-related challenges in clinical rotations and beyond. Furthermore, we designed this study as the first phase of a longitudinal evaluation, with plans to follow this cohort through their fourth year (M4) to assess how their attitudes toward RDs evolve over time and to solicit their recommendations for enhancing RD education within the preclinical curriculum. This longitudinal perspective aims to inform iterative improvements in medical education tailored to students’ developmental stages.

We employed a moderated Q&A format featuring an RD clinical care provider and three patients and their families with Abetalipoproteinemia, Tay Sachs, Prader Willi, and Gaucher—conditions chosen for their metabolic, neurodegenerative, and genetic diversity, based on local expert availability and willingness to share lived experiences. Since post-panel surveys contributed 1 point to the biochemistry course grade, we included SMP students alongside their M1 classmates to ensure fairness in earning this credit, broadening exposure to these critical perspectives across both groups.

We administered a pre-panel and post-panel survey via Qualtrics (Provo, UT) to assess their understanding of RD prevalence, patient and family impact, and their comfortability in interacting with to RD patients and families before and after attending the panel. Completion of surveys was optional, with students earning 1 point toward their course grade per submitted survey as an incentive to encourage participation without coercion. The pre-panel survey, distributed one week prior, included 17 multiple-choice and Likert-scale questions, while the post-panel survey, given immediately after, had 13 questions of the same format, focusing on knowledge gains and attitude shifts (see Supplemental Section for complete list of questions).

We gave students preparatory reading materials designed by the course director and instructed them to read the materials after taking the pre-panel survey. We asked students to identify appropriate resources for RD patients and providers. The reading materials include a brief introductory page on enzymatic defects in lysosomal storage diseases, a detailed 13-page NORD report covering nearly 50 lysosomal storage disorders with expert insights on symptoms, causes, and treatments, and a 7-page Osmosis.org notes document outlining glycogen storage diseases (types I–V), their pathology, diagnosis, and management, focusing on metabolic enzyme deficiencies. These documents are included in the Supplements.

We analyzed survey results using the R Project for Statistical Computing: R version 4.2.3 (2023-03-15). The pre-panel and post-panel survey datasets were combined into a single cohort for analysis using R (R Markdown document available in Supplementary materials). Responses to identical questions across both surveys were aligned and pooled directly, leveraging their consistent scales and response options. No transformations were applied prior to combining the data, given the structural similarity and shared target population of the surveys, which ensured comparability. This quasi-experimental pre–post design assessed immediate learning outcomes without a control group, prioritizing curriculum-wide feasibility over experimental control. Descriptive statistics, including means, medians, and quartiles, were calculated for the pooled dataset.

The Georgetown University Institutional Review Board approved the assessment of students under IRB ID: STUDY00006817 on 8 August 2023.***

### Scoping review

Our scoping review involved a systematic search of MEDLINE, Embase, ERIC (all through Ovid), and Web of Science, conducted on 7 July 2023 and included all years.

**Fig. 1:**
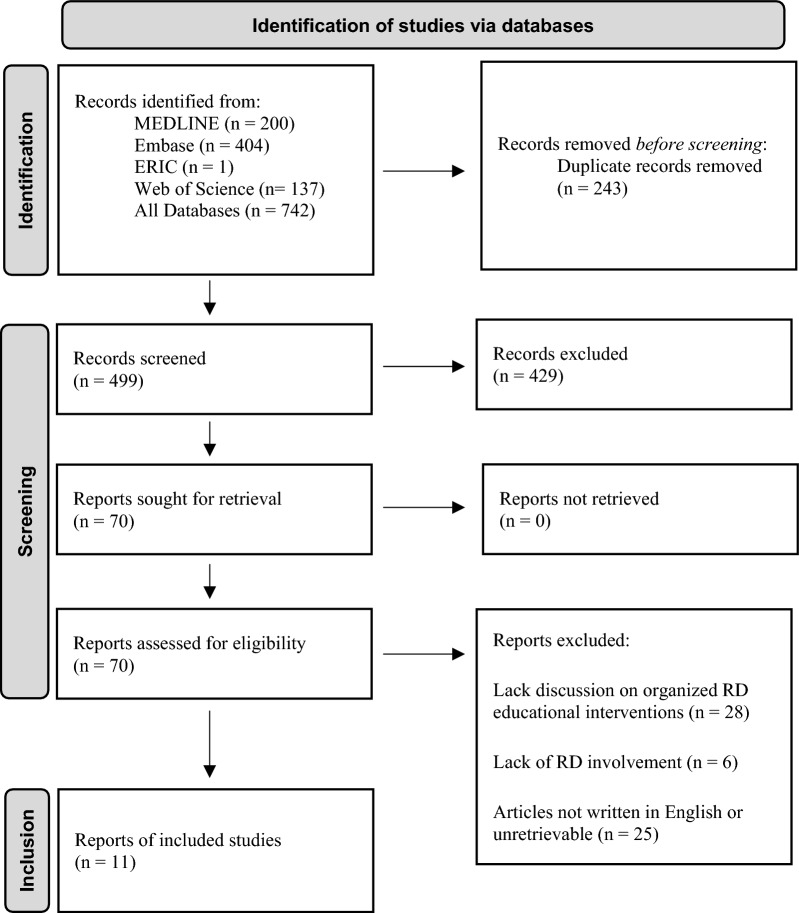
PRISMA flow diagram. Systematic scoping review flow diagram detailing the databases and the number of articles retrieved from each. Reasoning for exclusion and number of excluded articles at each step of the screening process, as well as the final number of articles in the scoping study. The scoping study was done at Georgetown University School of Medicine, in Washington, D.C., in July 2023

Search terms included keywords and database specific subject headings for the concepts rare diseases and undergraduate medical education (see Appendix/Supplement for the full reproducible search strategies). Inclusion criteria focused on peer-reviewed English-language studies of RD integration into undergraduate medical curricula, excluding non-educational or unpublished works (Table 1), to ensure relevance and accessibility. Using Rayyan [11], our team members (AP and RZ) independently evaluated 70 studies identified for full-text review and analyzed the interventions’ approaches, outcomes, and effectiveness. We resolved discrepancies through consultation with other team members (EW, SH, and RY) until we achieved consensus; however, this step was ultimately unnecessary as no disagreements occurred. The review process included 11 relevant articles into the scoping review (Fig. 1). This process aimed to identify scalable RD education strategies, informing recommendations for global medical curricula.

**Table 1:** Inclusion and exclusion criteria for scoping review methodology

Inclusion criteria	Exclusion criteria
Study investigates method(s) to include RD into medical education curriculum Specifically discusses *implementation* of education Include foreign language sources (exclude later if no translation) Include residency/GME training results	Study does not investigate method(s) to include RD into medical education curriculum Raising awareness for the need for RD education Sources focusing on pathology

The criteria were used during the screening process for articles addressing RD education in medical school. The scoping study was done at Georgetown University School of Medicine in Washington, D.C., in July 2023

## Results

### RD panel event

The pre-panel and post-panel surveys were distributed to a total of 284 students via an anonymous link shared through an email announcement. The anonymity of the link ensured that submissions could not be tracked to individual participants, allowing for candid responses. However, the total number of responses recorded for individual questions varied, ranging from 269 to 290. This discrepancy in response numbers can be attributed to two primary factors. First, the higher number of responses (exceeding the total number of participants) is likely due to some students submitting the survey multiple times, as the anonymous link did not restrict duplicate submissions. Second, the lower number of responses for certain questions is a result of the survey design, which allowed participants the option to skip questions they preferred not to answer. Consequently, some students provided responses to only a subset of the questions before submitting the survey. These factors account for the variation in response counts across the survey items (Table 2).

**Table 2:** Scoping summary of current RD teaching methodologies and interventions at U.S. and international medical schools

Study ID	Country (region)	Sample	Aim	Intervention description	Outcome	Recommendations
Morgenthau et al. [13]	Canada, USA	N = 334	To assess the long-term impact of the RARE Compassion Program on medical student interest in RDs	The RARE Compassion Program matched medical students with RD patients for eight months, requiring three in-person or virtual meetings per patient. Students could participate in an essay contest for a scholarship	12 out of 334 students completed a post-program survey, rating it 4.27/5, with 91.3% recommending it and 50.0% advocating for more RD discussions in medical education	Use peer-to-peer endorsement to raise program awareness among medical students. RD education should extend beyond medical students focused on primary care
Al-Jasmi et al. [19]	Worldwide (Canada, USA, Russia, China, Japan, UK, Australia, EU)	N = 12,000 users per month Feedback response 70–75%	To develop and assess a computer-based Hunter disease training program for pediatric and genetic residents	Researchers developed an interactive program that guides users in diagnosing and treating virtual Hunter disease patients, integrating a peer-reviewed textbook, guiding questions, and self-evaluation	70.0–75.0% of ~ 48,000 users responded to a feedback survey, expressing satisfaction and suggesting features like bookmarking and word-search to improve the program on Hunter disease	Virtual clinics can be used as a successful learning tool for other RDs beyond Hunter disease
Bai et al. [18]	China	N = 294	To assess medical students'learning and perceptions of rare and atypical disease cases in a problem-based curriculum	Students learned to differentiate common and RDs using a problem-based curriculum. The last four cases focused on one disease type, with all groups using the same learning style	Students studying RD reported the highest satisfaction, finding cases challenging, relevant, and beneficial for clinical skills, with a strong desire to continue learning about RDs	Encourage student engagement in RD education by appealing to their intellect and morality, integrating case reports in problem-based curricula, and balancing rare and common disease education to maintain diagnostic skills
Sanges et al. [21]	France	N = 38	To assess the educational impact of role-play simulation and patient educators in identifying"red flags"in common and rare presentations of Raynaud's phenomenon	A workshop taught RD triaging using role-play simulations of idiopathic RP and rare SSc. Groups of students, after a related lecture, split into physician actors and observers to perform medical tasks	Workshop participants scored higher on an SSc exam than non-participants and reported greater confidence, low stress, and strong satisfaction, with all recommending the workshop	Role play simulations and patient educators effectively taught clinical"red flags"and served as successful lecture supplements, with long-term interventions recommended
Jerrentrup et al. [20]	Germany	N = 157	To examine the long-term effects of House MD seminars on medical students'interest and knowledge in RDs	An extracurricular seminar series used House MD episodes for discussions on biomedical topics, patient safety, and diagnosis. Students rated their seminar experience	69.9% of students found incorporating House MD in seminars enhanced learning, with 89.7% reporting improved concentration and 86.7% better participation	Fun is an effective motivator, and carefully selected medical TV shows can be an innovative and engaging way to teach RDs to medical students
Greenberg et al. [23]	Israel	N = 5	To assess the effectiveness of short HSCR training on pathology residents'diagnostic accuracy and confidence using an AI decision support system	An AI-based decision support system (DDS) for diagnosing Hirschsprung disease (HSCR) was tested by pathologists on 1,704 images, with a pre- and post-training analysis using the DDS	DDS training improved HSCR diagnosis accuracy and pathologists'confidence while reducing reliance on expert consultations	AI-based programs are efficient and effective in assisting users with identifying RDs like HSCR but are also susceptible to false positives, causing the potential for overconfidence
Jonas et al. [14]	Poland	N = 270	To evaluate the baseline knowledge of RDs among medical, pharmacy, and health sciences students and the impact of targeted education	A 30-h elective on RDs covered various medical areas and included patient case reports, administered to a randomized intervention group	The intervention group showed higher accuracy in identifying RD characteristics and improved RD identification, with ALS, sarcoidosis, and cystic fibrosis most often correctly identified	Students'knowledge of RDs is low regardless of which training year they are in, so targeted education serves as a successful intervention for improving RD knowledge while standard education is insufficient
Bean et al. [17]	USA (Georgia)	N = 140	To assess first-year medical students'ratings of a case-based virtual lab on genetic testing for its educational value	Students completed virtual labs on genetic testing for five RDs after lectures and workshops. They selected tests, analyzed results, and practiced patient communication	92.0% of respondents approved of the virtual labs, with 88.0% believing they met learning objectives, and 94.0% noting their usefulness and effectiveness	To maximize the benefits of interventions, provide background knowledge, use virtual simulations with diverse cases, design open-ended scenarios for critical thinking, and maintain small groups for effective collaboration
Kestler et al. [16]	USA (Colorado)	N = 29	To evaluate the educational impact of a severe malaria simulation for 3rd-year medical students, residents, and nurses	A severe malaria simulation was conducted for medical students and emergency medicine staff and repeated five times after an initial pre-test on the same case	Survey respondents rated mannequin learning and simulation as highly effective, with an 85.0% accuracy rate on severe malaria knowledge post-simulation	Role-playing simulations are effective to both participants and observers’ short-term learning and have strong approval from students
Quick et al. [15]	USA (Ohio)	N = 24	To understand the effects of simulation training on medical students'interest and knowledge of malignant hyperthermia	Students engaged in an independent simulation on malignant hyperthermia after anesthesiology training, followed by a debrief on identification, management, and resources	Students were satisfied with the simulation, learning the importance of collaboration and urgency in managing malignant hyperthermia, despite initial hesitation to seek expert advice	Pre- and post-tests are essential for analyzing the impact of RD education interventions, and simulation training can be applied to RDs beyond malignant hyperthermia
Lane and Black [22]	USA (Virginia)	N = 44	To determine the educational benefits of using 3D-printed models to teach rarely presenting craniofacial pathologies	All students viewed a 25-min lecture on craniosynostosis, with a test group using 3D printed models. Pre- and post-module quizzes were given to assess learning	The test group reported better anatomical and surgical understanding, but no significant difference in quiz scores was found between the test and control groups, though both improved post-lecture	3D models can supplement learning experience for craniofacial pathology

The scoping study was done at Georgetown University School of Medicine in Washington, D.C., in 2023

We were first curious about students’ prior RD exposure. Among the 290 responses in the pre-panel survey, nine (3.1%) students indicated they have a personal RD diagnosis, and 40 (13.8%) indicated they have a family member with an RD diagnosis. 89 (30.7%) students participated in RD research and/or RD volunteering experiences in the past. We found that among 270 responses in the post-panel survey, 168 (62.2%) students rated the panel as “Extremely helpful,” and 189 (70.0%) said they now “care much more” about RDs. 163 (60.4%) students also believe there should be more RD content in medical education.

**Fig. 2:**
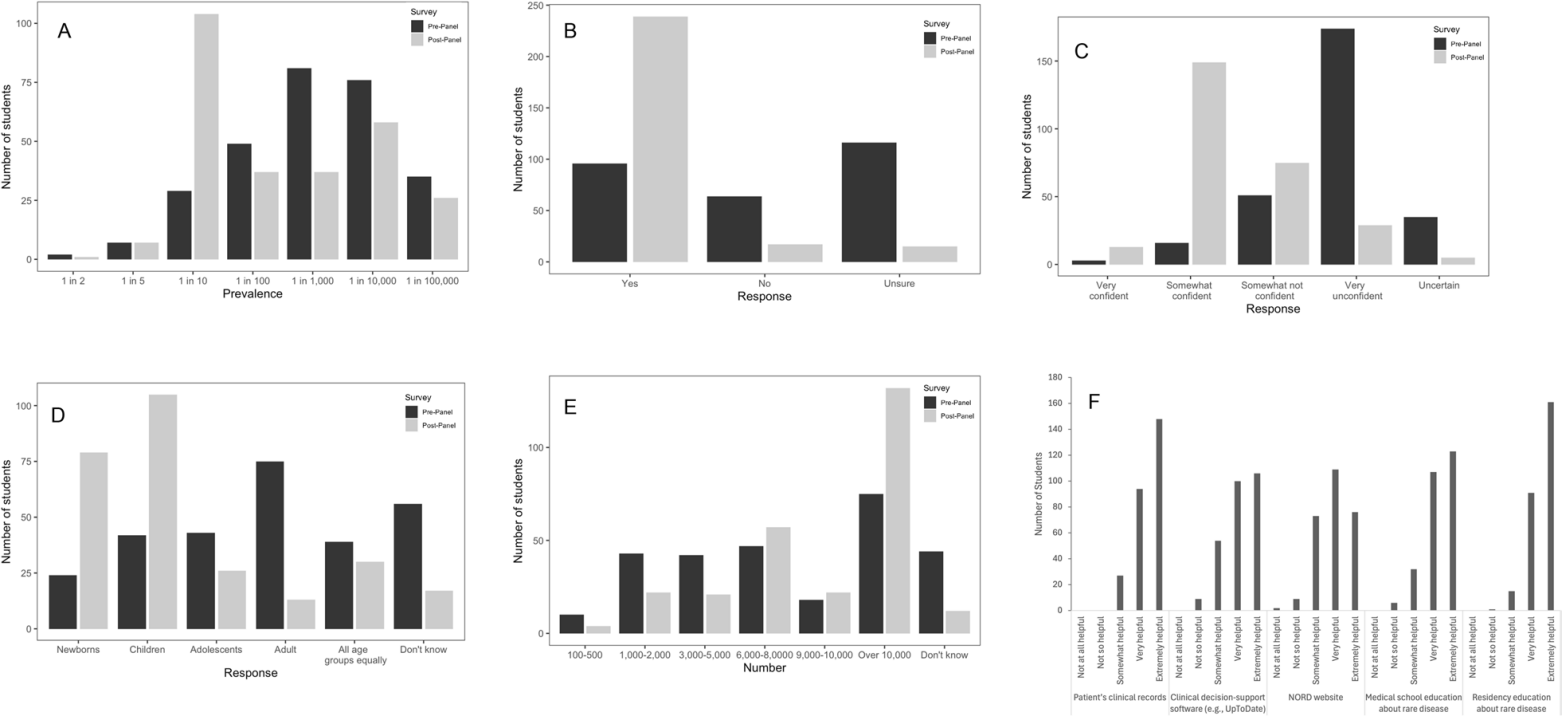
GUSOM first-year medical students’ responses in surveys administered before and after they attend the interactive rare disease panel event. **A** Impression of prevalence of all RDs combined. **B** Impression of RD as a major public health problem. **C** Confidence in caring for RD patients. **D** Impressions of age at diagnosis. **E** Impressions of estimated number of RDs. **F** Students’ identification of useful resources for RD education. Pre-panel survey *n* =290. Post-panel survey *n* =269. The surveys were conducted at Georgetown University School of Medicine, Washington D.C. in August, 2023

We were also interested in how the panel impacted students’ understanding of RDs. After attending the panel of RD patients and care providers, more medical students better understood RD prevalence, demonstrated by an increase in correct identification of RD prevalence. Before attending the panel, 10.3% (29/218) of respondents correctly chose “1 out of 10 Americans have an RD.” After attending the panel, 38.5% (104/270) of respondents chose the correct answer (p-value = 2.32e-07) (Fig. 2A). Students were also more likely to recognize that RD poses significant public health concerns. When asked, “Do you consider RDs a major public health problem?”, 34.8% (96/276) responded “Yes” before attending the panel. This number increased to 88.2% (239/271) after students attended the panel (p-value < 2.2e-16) (Fig. 2B). After attending the panel, students’ ability to correctly identify the average time of diagnosis for RDs increased from 23.7% to 68.2% (p-value < 2.2e-16) (Fig. 2D). Their awareness that there are over 10,000 recognized RDs rose from 26.9% to 48.9% (p-value = 2.28e-09) (Fig. 2E).

It is important to understand how gaining more understanding about RDs might translate into students’ clinical behavior. Prior to the panel, the majority (62.4%) of students rated their confidence in caring for RD patients as “Very Unconfident.” After the panel, the majority (55.0%) answered, “Somewhat Confident” (p-value < 2.2e-16) (Fig. 2C).

Finally, we were interested in understanding what resources medical students feel would be most helpful in their care for future patients with RDs. Students identified each resource as “Not at all helpful,” “Not so helpful,” “Somewhat helpful,” “Very helpful,” and “Extremely helpful.” The resource that the highest number (60.1%) of students identified as “Extremely helpful” was “Residency education about RD” (Fig. 2F). The second and third most identified as “Extremely helpful” resources were “Patient’s clinical records” and “Medical school curriculum about RD” at 55% and 45.7%, respectively (Fig. 2F).

### Systematic scoping review on RD medical education

We conducted a systematic scoping review to evaluate the current state of RD education in medical schools and identify effective teaching methods. From 499 articles initially identified, 11 passed our inclusion and exclusion criteria described in Fig. 1 for final analysis. Publication dates ranged between 2010 and 2023, and countries of origin included the U.S. (*n* =4, 36.4%), China (*n* =1, 9.1%), France (*n* =1, 9.1%), Israel (*n* =1, 9.1%), Germany (*n* =1, 9.1%), Poland (*n* =1, 9.1%), and multiple countries (*n* =2, 18.2%). U.S. institutions included Emory University School of Medicine, the University of Colorado School of Medicine, the University of Toledo Medical Center, and the University of Virginia School of Medicine. The reach of these studies ranged from 19 to 334 students enrolled in MD, DO, or international medical programs.

Interventions included lectures (*n* =7, 63.6%), case studies (*n* =6, 54.5%), simulations (*n* =4, 36.4%), small group discussions (*n* =4, 36.4%), technology-based interventions (*n* =3, 27.3%), workshops (*n* =2, 18.2%), mentorship programs (*n* =1, 9.1%), anatomical models (*n* =1, 9.1%), and medical television drama series (*n* =1, 9.1%). Eight studies utilized more than one intervention at once. In designing these interventions, the opinions or participation of RD patients (*n* =3, 27.3%) or RD caregivers (*n* =1, 9.1%) were scant, but the expertise of RD healthcare professionals (*n* =9, 81.8%) was abundantly incorporated.

All educational content pertained to RDs but ranged broadly in disease specificity. Specific RDs were the focus of seven studies and included Hunter disease; paroxysmal nocturnal hemoglobinuria of the hematologic system; hepatolenticular degeneration of the digestive system; systemic sclerosis of Raynaud’s phenomenon; Hischsprung disease; Duchenne muscular dystrophy; classic and Duarte galactosemia; Lynch syndrome; mosaic trisomy 20; severe malaria; malignant hyperthermia; and craniosynostosis. Four studies focused on RD generally. Most studies analyzed outcomes after one round of intervention (*n* =9, 81.8%) instead of over multiple cycles (*n* =2, 18.2%). All studies that assessed student satisfaction reported positive responses (*n* =7, 63.6%) and student knowledge (*n* =5, 45.5%) reported significant increases post-intervention.

## Discussion

In 2023, Rohani-Montez et al. shed light on striking misconceptions among physicians in the U.S. regarding RDs [12]. They found that only 8% of physicians accurately identified the defining threshold for RDs as any condition affecting < 200, 2000 individuals, with 47% holding a mistaken belief that 1 in 100,000 Americans had an RD [12]. Contrary to this misconception, RDs affect 1 in 10 Americans [1], highlighting a gap in medical education and the urgent need for better training to improve awareness among medical students and physicians.

To address this educational gap, we implemented an RD patient panel that offered valuable insights into the effectiveness of integrating patient perspectives into RD education. We found that learning through interactive conversations with RD patients, their families, and their caregivers positively impacted medical students’ understanding of and attitudes toward RDs. After attending the panel, students were more likely to correctly identify the prevalence of RD (Fig. 2A), the estimated number of RD (Fig. 2E), and the common age of RD diagnosis (Fig. 2D). Students were also more likely to agree that RD is a public health problem (Fig. 2B) and felt more confident in caring for RD patients (Fig. 2C). Encouragingly, students displayed an interest in long-term RD learning by indicating residency education about RD as the highest-rated answer (Fig. 2F). Overall, students received our patient panel very well. Students not only gained concrete knowledge on RD as a public health problem and challenges that RD patients face in their care journey, but our students also became more enthusiastic about learning about RD longitudinally.

While some educational interventions cited in the scoping review, such as extended courses, offer in-depth exploration of RD complexities, the purpose of our panel was distinct: to serve as an accessible entry point that humanizes rare diseases for first-year students. By featuring real patients and caregivers, the panel aimed to remove the abstractness of RDs, cultivate empathy, and inspire advocacy—qualities essential for students as they encounter RD patients in their future careers. Far from being a standalone solution, this brief intervention acts as a springboard, preparing students with a compassionate mindset for the longitudinal academic journey of RD education, which continues through system-based preclinical lectures and clinical experiences over their training years. Our results, showing significant improvements in students’ attitudes, knowledge, and confidence (Fig. 2A–C), affirm the panel’s effectiveness within this limited scope, complementing rather than competing with more comprehensive approaches.

The positive impact that we found was like that observed in other studies that introduced medical students to RD through interactive learning. For example, Morgenthau et al. (2022) found that students who participated in the RARE Compassion Program demonstrated a higher understanding of RD and a continued desire to learn more about RD [13]. Jonas et al. (2017) showed improved RD diagnosis accuracy by students who participated in an elective RD course [14]. Quick et al. (2017) showed that students who participated in simulation studies of malignant hyperthermia identified collaboration and urgency as key to diagnosis and treatment [15]. We also found that active learning approaches enhanced students’ clinical skills and patient interaction abilities. For example, role-playing simulations using mannequin [16], virtual collaboration activities in choosing RD diagnostic tools and interpreting results [17], problem-solving-based curriculum [18], interactive computer-guided diagnosis and treatments of Hunter disease [19], the use of the television medical TV show “House MD” as a teaching tool [20], immersive workshops to identify RD “red flags” [21], and hands-on learning with 3D models for craniofacial pathology [22] all demonstrate increased student competency in RD understanding.

The scoping review of 11 studies on teaching RDs in medical schools reveals promising beginnings for innovative educational approaches and their impact on student learning outcomes. Hands-on learning programs and simulation-based training emerged as effective tools in medical education, significantly enhancing diagnostic accuracy, confidence, and essential skills crucial for future clinical practice [13, 15, 21]. For instance, the RARE Compassion Program facilitated partnerships between medical students and RD patients, fostering empathy and understanding while providing firsthand exposure to real-world patient interactions [13]. Similarly, workshops utilizing role-play simulations, such as those conducted by Sanges et al. (2020), were instrumental in improving diagnostic skills by teaching students to recognize “red flags” in triaging patients with suspected RDs [21].

Excitingly, the integration of technology, including artificial intelligence (AI) decision support systems and virtual laboratories, has shown promise in enhancing student engagement and knowledge acquisition [23]. Greenberg et al. (2023) developed an AI-based decision support system for diagnosing Hirschsprung disease, which significantly improved the accuracy of pathological diagnoses compared to traditional methods [23]. Additionally, as Bean et al. (2011) demonstrated, virtual laboratories provide students with hands-on experience in selecting appropriate genetic tests for RDs, enhancing their practical skills and decision-making abilities in genetics [17].

Incorporating these innovative approaches into medical education enriches the learning experience and equips students with the necessary tools to excel in clinical practice, ultimately improving patient care outcomes. Due to the diverse range of RDs, learning curricula should be innovative and flexible to help students develop clinical skills and empathy when caring for RD patients. While our panel event emphasized metabolic disorder care and diagnostic journeys, the scoping review encompassed various RDs ranging from hematologic disorders to digestive system conditions. This diversity underscores the necessity of comprehensive curricula that expose students to various RDs. Technology and interactivity play pivotal roles in both contexts, highlighting the importance of engaging students directly with patients, families, and caregivers and leveraging technologies, such as decision support systems and simulations. Striking a balance between simulated scenarios and real-life patient experiences, such as those provided by the GUSOM panel and the RARE Compassion Program, ensures students develop core interpersonal and reasoning competencies as recommended by AAMC in the 2005 Recommendations for Clinical Skills Curricula for Undergraduate Medical Education [8]. By learning about RDs, students will develop a deeper understanding of diverse medical conditions, enhance their diagnostic skills, and improve their ability to provide empathetic, informed care to patients with RDs.

In our scoping review, we noted a lack of patient-centered learning experiences that incorporate patients’, families’, and caregivers’ voices in the planning of RD in medical education. Through positive feedback and improvements in students’ awareness of RD after the interactive panel at GUSOM, we believe having the patients, their families and their caregivers at the center of the learning experience is crucial for meaningful engagement and long-term interest in RD education. Integrating more patient-led discussions and role-playing simulations into the curriculum could provide a deeper, more empathetic understanding of the challenges faced by RD patients. Such methods could be evaluated through feedback surveys and performance assessments to continually adjust and improve their impact. Curricula should also reflect global health disparities, preparing students to manage RDs in varied contexts—from high-resource settings like the U.S. to LMICs, where 30% of children with RDs die before age 5 due to delayed diagnosis and lack of treatment. This patient-centric approach could be further enhanced by establishing multi-center RD registries and specialized referral clinics, which would aggregate data for studying RD patterns and provide hands-on training with expert mentorship, improving both education and patient care worldwide. It would also be beneficial to optimize the range of RDs covered in educational programs, as emphasized by the diverse conditions explored in the panel events and scoping reviews, ensuring that students gain a broad perspective on the variety of presentations and complexities associated with RDs. This holistic approach should be coupled with ongoing CME opportunities that reinforce and update knowledge throughout a student and physician’s career, maintaining a high level of competency in diagnosing and treating RDs [24].

Additionally, continuous innovations and long-term learning are crucial to improve the effectiveness of discussed strategies. While patient-led discussions, simulation-based training, and interactive technologies show promise in enhancing student engagement and learning outcomes, we call for further exploration of these approaches to optimize their effectiveness. For instance, expanding on interactive technologies and incorporating patients’ suggestions in virtual laboratories and decision support systems could help refine their design, improve the simulation of real-life scenarios, and enhance the practical application of learned knowledge in clinical settings [17, 22]. Continuous exposure to RD education is another target for improving students’ clinical skills. One recommendation for students and physicians to conveniently access RD education is through NORD’s Online Continuing Medical Education (CME) courses [24]. This option offers accredited online courses developed in partnership with PlatformQ Health, showing that 92% of participating clinicians reported a positive impact on their practice, and 90% noted improved patient experiences and outcomes through learning sessions that incorporate patient voices [25]. These courses provide accessible and engaging digital content focusing on disease-specific and general RD education, which is ideal for equipping healthcare providers to identify symptoms and review treatment options for RDs.

Focusing on first-year students in our panel event allowed us to establish a baseline of RD awareness at the outset of their medical education, a critical step given their limited foundational knowledge at this stage. This choice supports our longitudinal goal to follow this cohort through their training, with a planned M4 survey to evaluate how their attitudes toward RDs evolve and whether they recommend adjustments to preclinical RD education to better prepare them for clinical rotations and beyond. Capturing these early perspectives also aids curriculum designers in identifying areas needing emphasis—such as prevalence or diagnostic challenges—ensuring that RD education builds progressively as students’ medical knowledge deepens.

Beyond enhancing educational curricula, our findings underscore the critical need for systemic improvements in RD care infrastructure. The establishment of multi-center registries for rare diseases, alongside specialized clinics dedicated to the referral and management of cases with suspected manifestations, would significantly bolster early detection and diagnosis. These resources would pool expertise from various centers, facilitating timely interventions and improving overall care for RD patients. Moreover, such registries and clinics would serve as invaluable educational tools, offering medical students and personnel access to aggregated data and real-world case experiences, thereby reinforcing classroom learning with practical exposure.

We would like to note some limitations present in our study. First, the evaluation of the panel event at GUSOM may not be generalizable to other medical schools across the country. Factors such as student demographics and their previous RD exposures, institutional resources, or curricula may play a role in students’ baseline understanding of RD and specific strategies for growth. GUSOM’s urban location, with access to tertiary hospitals and RD communities, facilitates collaboration with RD patients and interactive teaching methods, such as Q&A panels. In contrast, medical schools with limited access to RD communities may find it more difficult to recruit patients and caregivers for such interactive learning experiences. Secondly, self-reported data from the pre-and post-panel surveys have the potential for response bias. Students may overstate their learning gains or refer to external sources to answer questions in the post-survey, potentially distorting the results’ accuracy. Thirdly, in our scoping review, the quality and heterogeneity of the relevant studies, which vary in their methodologies, sample sizes, and educational interventions, may have been limiting. During the review process, we may have missed relevant unindexed or non-English studies. Finally, our study does not assess long-term knowledge retention or changes in clinical practice behaviors; this is an area of continued study. We hope to implement the RD panel event at GUSOM annually and survey each cohort of students as they progress through the years at medical school and into residency.

### Recommendations for medical education

Considering these observations collectively, we propose several strategies for medical schools to enhance the integration of RD education into their curricula. Schools should incorporate patient-led discussions and role-playing simulations to foster empathy and provide a deeper understanding of the challenges faced by RD patients. They should also utilize technology, such as AI-based decision support systems and virtual laboratories, to improve diagnostic accuracy and engage students in practical, hands-on learning experiences. Lastly, they could offer continuous education opportunities, such as online CME courses, to ensure long-term knowledge retention and keep healthcare providers updated on the latest advancements in RD care. These strategies should be tailored to prepare students for a global healthcare landscape, incorporating case studies and technologies that reflect the challenges of RD care in LMICs, where resources are limited. Additionally, medical schools should advocate for establishing multi-center RD registries and specialized clinics, enhancing education by providing access to pooled data and expert-led clinical exposure while facilitating early detection and management of RDs. Table 3 summarizes our proposed strategies [24].

**Table 3:** Recommended strategies for medical schools to integrate RD education into curricula

Recommendation	Description	Examples
Integrate Diverse and Adaptable Teaching Approaches	Combine various teaching methodologies, such as interactive Q&A panels with RD patients and problem-based learning (PBL), to create a comprehensive RD education program. Recognize the importance of adaptability by developing a dynamic curriculum that evolves with emerging technologies, educational research, and the evolving landscape of RD knowledge	RD patient panel (this manuscript) Partnership programs with RD patients and caretakers [13] Problem-based learning [18, 19] Case reports [14, 18, 19] Scenario-based simulation [15, 17] Role-playing simulation [16, 21] 3D anatomical modeling [22] Technology-based interventions [16, 17, 19, 23] Popular culture-based interventions [20]
Develop Comprehensive Curricula	Design curricula that expose students to a variety of RDs, ensuring a broad understanding of the challenges associated with different conditions. Consider incorporating real-life case studies to provide practical insights into RD care and diagnostics	RD patient panel (this manuscript) Partnership with any volunteer RD patient [13] Differentiating common diseases with typical symptoms, common diseases with atypical symptoms [19] RD elective class organized by body system [14] Genetic testing for Duchenne muscular dystrophy, Galactosemia, Lynch syndrome, and Mosaic Trisomy 20 [17] Case reports [14, 18, 19]
Leverage Technology and Interactivity	Embrace technology and interactive formats to enhance student engagement. Utilize decision support systems, virtual clinics, and simulations to create dynamic learning experiences that closely simulate real-world scenarios	Virtual labs [17, 19] Artificial intelligence decision support system for diagnosis [23] Electronic mannequin simulation [16]
Strike a Balance Between Simulation and Real-life Experience	Integrate simulated scenarios that closely mimic real-world experiences while ensuring students have opportunities for direct patient, family, and caregiver interactions. This balanced approach fosters the development of both technical and interpersonal skills	RD patient panel (this manuscript) Scenario-based simulation of malignant hyperthermia [15] Scenario-based simulation of genetic testing for five RDs [17] Role-playing simulation of severe malaria [16] Role-playing simulation of triaging idiopathic Raynaud's phenomenon and systemic sclerosis [21]
Explore the Role of AI and Decision Support Systems	Investigate the potential of artificial intelligence (AI) and decision support systems in enhancing diagnostic capabilities. Provide students with exposure to cutting-edge technologies that are increasingly relevant in RD diagnostics	AI decision support system for diagnosis of Hirschsprung disease in ganglia images [23]
Continuing medical education with the National Organization for Rare Disorders	Partner with NORD and PlatformQ Health to create an accredited online video library that provides healthcare providers with essential information on identifying and treating RDs. This resource should feature expert-led, engaging content accessible through leading educational platforms to effectively address knowledge gaps	Value of collaborating with non-healthcare professional RD stakeholders [13]

Synthesis of the current landscape of RD in the Scoping Study and data from the RD Patient Panel at Georgetown School of Medicine illuminates possible strategies for improving RD education in medical school. The scoping study was done at Georgetown University School of Medicine in Washington, D.C., in 2024

## Conclusion

In conclusion, addressing misconceptions and enhancing awareness among physicians and medical students regarding RD is paramount, given their prevalence and impact on patient care. Experiential learning programs, simulation-based training, and technology integration emerged as effective strategies to improve diagnostic accuracy, confidence, and essential skills crucial for future clinical practice. However, challenges such as the need for standardized assessment tools and the optimization of educational methodologies persist. Moving forward, a nuanced and adaptable approach that combines diverse teaching methods, comprehensive curricula, and ongoing CME opportunities is essential to ensure that current and future medical professionals have the tools to diagnose and treat RDs effectively throughout their careers. This approach must extend beyond U.S.-centric frameworks to equip physicians for the global burden of RDs, affecting 300 million individuals worldwide, particularly in LMICs where marginalized populations face heightened barriers. Supporting this, the establishment of multi-center RD registries and specialized clinics would further enhance education by providing data-driven insights and clinical exposure, while improving early diagnosis and care coordination.

## Supplementary Information

Additional file 1.

Additional file 2.

Additional file 3.

Additional file 4.

Additional file 5.

Additional file 6.

## Data Availability

The data included in this manuscript were presented at the National Organization for Rare Disorders Breakthrough Summit 2023 and 2024. No specific data from outside sources were used in this study. The datasets generated and analyzed during the current study are available from the corresponding author upon reasonable request.
