# Supplementary figures and images for: Rare disease education in medical schools: patient-centered and innovative strategies
Source: Orphanet J Rare Dis. 2025 Nov 20;20:596. doi: 10.1186/s13023-025-03771-8 (PMC12632075; doi:10.1186/s13023-025-03771-8)

## Enzymatic Defects in Lysosomal Storage Diseases

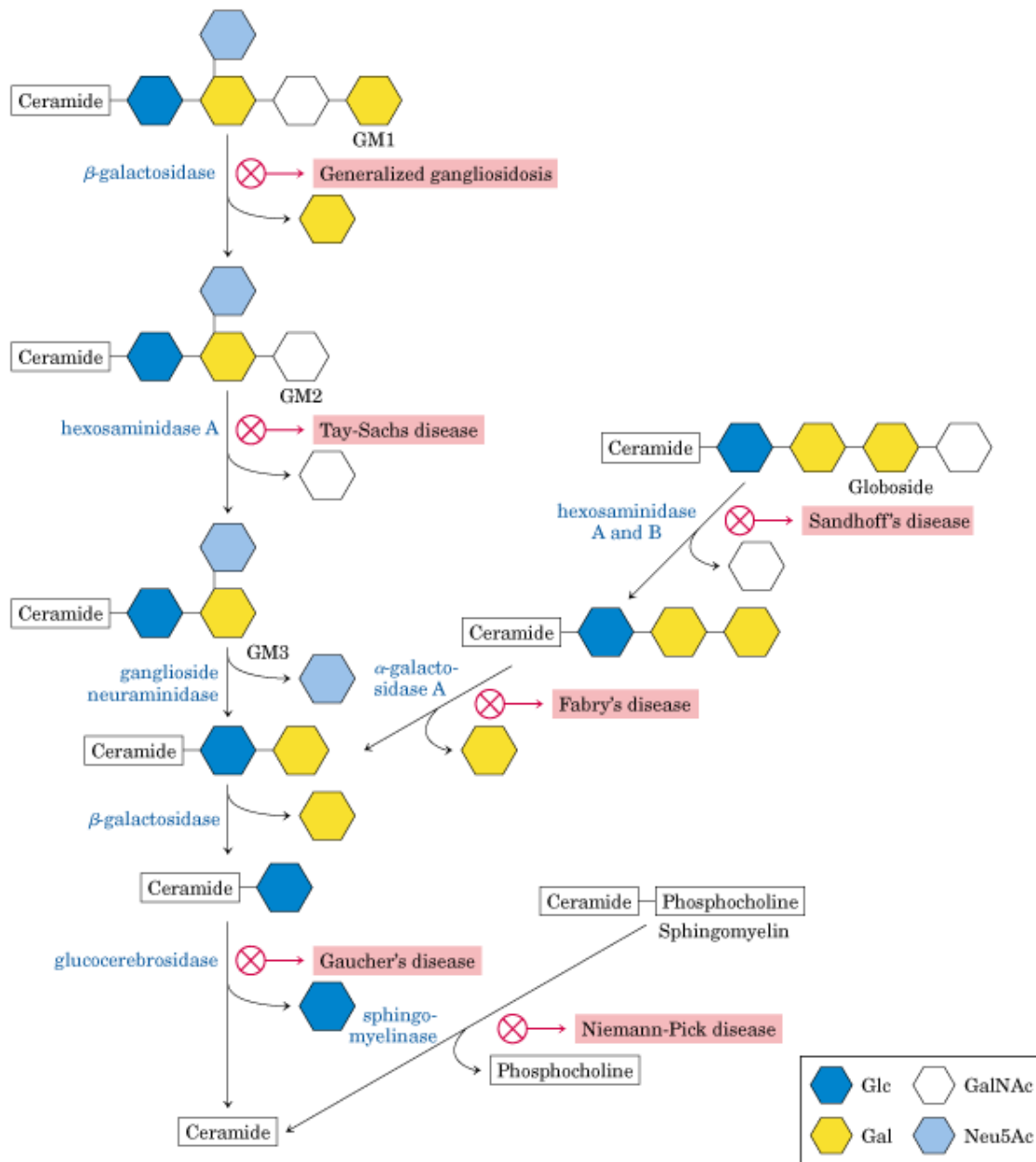

Supplement: Supplementary file 1 — Additional file 1. [file 13023_2025_3771_MOESM1_ESM.pdf]
