# Supplementary material for: Rare disease education in medical schools: patient-centered and innovative strategies
Source: Orphanet J Rare Dis. 2025 Nov 20;20:596. doi: 10.1186/s13023-025-03771-8 (PMC12632075; doi:10.1186/s13023-025-03771-8)
Supplement: Supplementary file 2 — Additional file 2. [file 13023_2025_3771_MOESM2_ESM.pdf]

Lysosomal Storage Disorders

rarediseases.org/rare-diseases/lysosomal-storage-disorders/

NORD gratefully acknowledges Joe T.R. Clarke, MD, PhD, Director of the Genetic Metabolic Disease Program at The Hospital for Sick Children in Toronto for assistance in the preparation of this report.

General Discussion

Lysosomal storage diseases are inherited metabolic diseases that are characterized by an abnormal build-up of various toxic materials in the body's cells as a result of enzyme deficiencies. There are nearly 50 of these disorders altogether, and they may affect different parts of the body, including the skeleton, brain, skin, heart, and central nervous system. New lysosomal storage disorders continue to be identified. While clinical trials are in progress on possible treatments for some of these diseases, there is currently no approved treatment for many lysosomal storage diseases.

Signs & Symptoms

Although the signs and symptoms vary from disease to disease in this group, symptoms occur in each case because of an enzyme deficiency that inhibits the ability of the lysosomes present in each of the body's cells to perform their normal function. The lysosomes function as the primary digestive units within cells. Their function is to break down complex components into simpler ones. Each cell has hundreds of lysosomes that degrade complex cellular components such as proteins (substrates) into simpler components. When this process does not take place, the substrate begins to accumulate in the cells. That is why these diseases are called "storage diseases". The symptoms of lysosomal storage disorders are generally progressive over a period of time.

This report gives an overview of lysosomal storage diseases as a group. For more specific information on any particular disease in this group, consult the NORD report on that topic.

Some lysosomal storage diseases and a few of their characteristic signs and symptoms are as follows:

Aspartylglucosaminuria: Patients appear normal for several months after birth and then present with recurrent infections, diarrhea, and hernias. Later, there may be a gradual

coarsening of facial features, an enlarged tongue (macroglossia) and enlargement of the liver (hepatomegaly).

Batten Disease: Batten disease is the juvenile form of a group of progressive neurological disorders known as neuronal ceroid lipofuscinoses (NCL). It is characterized by the accumulation of a fatty substance (lipopigment) in the brain, as well as in tissue that does not contain nerve cells. Batten disease is marked by rapidly progressive vision failure (optic atrophy) and neurological disturbances, which may begin before eight years of age. Occurring mostly in families of Northern European Scandinavian ancestry, the disorder affects the brain and may cause deterioration of both intellect and neurological functions.

Cystinosis: The early signs of this disorder typically involve the kidneys and the eyes. Excessive storage of the amino acid cystine in all cells of the body results in impaired kidney function, increased sensitivity to light, and marked growth retardation. There are infantile (the most common and most severe), juvenile, and adult forms, each with associated symptoms.

Fabry Disease: The symptoms of Fabry disease usually begin during early childhood or adolescence but may not become apparent until the second or third decade of life. Early symptoms include episodes of severe burning pain in the hands and feet. Other early signs may include a decrease in sweat production, discomfort in warm temperatures, and the appearance of a reddish to dark blue skin rash, especially in the area between the hips and knees. These skin lesions may be flat or raised, and some people may not have them at all.

Gaucher Disease Types I, II, and III: Gaucher disease is the most common type of lysosomal storage disorder. Researchers have identified three distinct types of Gaucher disease based upon the absence (type I) or presence and extent of (types II and III) neurological complications. Most affected individuals have type I, and they may experience easy bruising, chronic fatigue, and an abnormally enlarged liver and/or spleen (hepatosplenomegaly). Gaucher disease type II occurs in newborns and infants, and is characterized by neurological complications that may include involuntary muscle spasms, difficulty swallowing and the loss of previously acquired motor skills. Gaucher disease type III appears during the first decade of life. Neurological complications may include mental deterioration, an inability to coordinate voluntary movements, and muscle spasms of the arms, legs, or entire body.

Glycogen Storage Disease II (Pompe Disease): Pompe disease has an infantile form and a delayed onset form. The delayed onset form may be further broken down into a childhood form and a juvenile/adult form. Patients with the infantile form are the most severely affected. Although these infants usually appear normal at birth, the disease presents within the first two to three months with rapidly progressive muscle weakness, diminished muscle tone (hypotonia) and a type of heart disease known as hypertrophic cardiomyopathy. Feeding problems and respiratory difficulties are common. The

childhood form presents during infancy or early childhood. Motor milestones may be delayed and some symptoms may resemble muscular dystrophy. The cardiac enlargement that is often present in the infantile form is seldom seen in the childhood form. The juvenile/adult form presents between the first and seventh decades as a slowly progressive muscle weakness or with symptoms of respiratory insufficiency. There is no cardiac involvement with this form.

GM2-Gangliosidosis Type I (Tay Sachs Disease): Two main forms of Tay Sachs disease exist: the classic or infantile form and the late-onset form. In individuals with infantile Tay Sachs disease, symptoms typically first appear between three and five months of age. These may include feeding problems, general weakness (lethargy), and an exaggerated startle reflex in response to sudden loud noises. Motor delays and mental deterioration are progressive. In individuals with the late-onset form, symptoms may become apparent anytime from adolescence through the mid-30s. The infantile form often progresses rapidly, resulting in significant mental and physical deterioration. A characteristic symptom of Tay Sachs disease, which occurs in 90 percent of cases, is the development of cherry red spots in the backs of the eyes. Symptoms of late-onset Tay Sachs disease vary widely from case to case. This disorder progresses much more slowly than the infantile form.

GM2-Gangliosidosis Type II (Sandhoff Disease): The first symptoms of Sandhoff disease typically begin between the ages of three and six months. The disease is clinically indistinguishable from GM2-Gangliosidosis Type I.

Metachromatic Leukodystrophy: Early signs and symptoms may be vague and gradual, making this disorder difficult to diagnose. Unsteadiness when walking is often the first symptom observed. Occasionally, the earliest symptom is developmental delay or deteriorating school performance. Over time, symptoms may include marked spasticity, seizures, and profound mental retardation.

Mucolipidosis Types I, II/III and IV: Mucolipidosis I, also known as sialidosis, has juvenile and infantile forms (sialidosis type I and sialidosis type II). Sialidosis type I usually becomes apparent during the second decade of life with the advent of sudden involuntary muscle contactions, the appearance of red sopts (cherry-red macules) in the eyes, and/or other neurological findings. Sialidosis type II may begin during infancy or later and is characterized by the same visual characteristics as sialidosis type I, as well as other symptoms such as mildly coarse facial features, skeletal malformations, and/or mild mental retardation. Symptoms of ML II, also known as I-cell disease, typically become apparent during infancy and include abnormalities of the skull and face, growth failure, and/or mental retardation. Type III, also known as pseudo-Hurler disease, is characterized by stiffness of the hands and shoulders with later development of carpal tunnel syndrome, deterioration of hip joints, scoliosis, and short stature. ML IV is characterized by mental retardation, greatly reduced ability in the acquisition of skills

requiring the coordination of muscular and mental activities, corneal clouding, retinal degeneration, and diminished muscle tone.

Mucopolysaccharide Storage Diseases (Hurler Disease and variants, Hunter, Sanfilippo Types A,B,C,D, Morquio Types A and B, Maroteaux-Lamy and Sly diseases): The MPS diseases are caused by disturbances in the normal breakdown of complex carbohydrates known as mucopolysaccharides. All of the MPS diseases have certain characteristics in common, which include deformities of the bones and joints that interfere with mobility and often cause osteoarthritis, especially of the large, weight-bearing joints. All of the MPS diseases except Sanfilippo disease interfere with growth, causing short stature.

Niemann-Pick Disease Types A/B, C1 and C2: Niemann-Pick disease is a group of inherited disorders related to fat metabolism. Certain characteristics common to all types include enlargement of the liver and spleen. Children with Niemann-Pick disease, types A or C, also experience progressive loss of motor skills, feeding difficulties, progressive learning disabilities, and seizures.

Schindler Disease Types I and II: Type I, the classical form, first appears during infancy. Affected individuals appear to develop normally until approximately one year of age, when they begin to lose previously acquired skills that require the coordination of physical and mental activities. Type II is the adult-onset form. Symptoms may include the development of clusters of wart-like discolorations on the skin, permanent widening of groups of blood vessels causing redness of the skin in affected areas, relative coarsening of facial features, and mild intellectual impairment.

Causes

In each case, lysosomal storage diseases are caused by an inborn error of metabolism that results in the absence or deficiency of an enzyme, leading to the inappropriate storage of material in various cells of the body. Most lysosomal storage disorders are inherited in an autosomal recessive manner.

Chromosomes, which are present in the nucleus of human cells, carry the genetic information for each individual. Human body cells normally have 46 chromosomes. Pairs of human chromosomes are numbered 1 through 22 and the sex chromosomes are designated X and Y. Males have one X and one Y chromosome and females have two X chromosomes. Each chromosome has a short arm designated “p” and a long arm designated “q”. Chromosomes are further subdivided into many bands that are numbered. For example, “chromosome 11p13” refers to band 13 on the short arm of chromosome 11. The numbered bands specify the location of the thousands of genes that are present on each chromosome.

Recessive genetic disorders occur when an individual inherits the same abnormal gene for

the same trait from each parent. If an individual receives one normal gene and one gene for the disease, the person will be a carrier for the disease, but usually will not show symptoms. The risk for two carrier parents to both pass the defective gene and, therefore, have an affected child is 25% with each pregnancy. The risk to have a child who is a carrier like the parents is 50% with each pregnancy. The chance for a child to receive normal genes from both parents and be genetically normal for that particular trait is 25%. The risk is the same for males and females.

All individuals carry four or five abnormal genes. Parents who are close relatives (consanguineous) have a higher chance than unrelated parents to both carry the same abnormal gene, which increases the risk to have children with a recessive genetic disorder.

Although most lysosomal storage disorders follow an autosomal recessive inheritance pattern, there are exceptions. Fabry disease and Hunter syndrome follow an X-linked recessive inheritance pattern. X-linked recessive genetic disorders are conditions caused by an abnormal gene on the X chromosome. Females have two X chromosomes but one of the X chromosomes is "turned off" and all of the genes on that chromosome are inactivated. Females who have a disease gene present on one of their X chromosomes are carriers for that disorder. Carrier females usually do not display symptoms of the disorder because it is usually the X chromosome with the abnormal gene that is "turned off". (However, it has been noted that some carriers of Fabry disease do experience significant clinical problems.) Males have one X chromosome and if they inherit an X chromosome that contains a disease gene, they will develop the disease. Males with X-linked disorders pass the disease gene to all of their daughters, who will be carriers. Males cannot pass an X-linked gene to their sons because males always pass their Y chromosome instead of their X chromosome to male offspring. Female carriers of an X-linked disorder have a 25% chance with each pregnancy to have a carrier daughter like themselves, a 25% chance to have a non-carrier daughter, a 25% chance to have a son affected with the disease, and a 25% chance to have an unaffected son.

The genes associated with many, but not all, lysosomal storage disorders have been identified. To learn more about the genetic locations associated with specific diseases, search the Rare Disease Database for reports on those topics.

Affected Populations

As a group, lysosomal storage diseases are believed to have an estimated frequency of about one in every 5,000 live births. Although the individual diseases are rare, the group together affects many people around the world. Some of the diseases have a higher incidence in certain populations. For instance, Gaucher and Tay-Sachs diseases are more prevalent among the Ashkenazi Jewish population. A mutation associated with Hurler syndrome is known to occur more frequently among Scandinavian and Russian peoples.

Diagnosis

Prenatal diagnosis is possible for all lysosomal storage disorders. Early detection of lysosomal storage diseases, whether before birth or as soon as possible afterward, is important because when therapies are available, either for the disease itself or for associated symptoms, they may significantly limit the long-term course and impact of the disease.

Standard Therapies

Treatment

There is no cure for lysosomal storage disorders, and there are not yet specific treatments for many of these diseases. However, progress is being made in the search for therapies, and there are treatments available for some lysosomal storage disorders that greatly improve the quality of life for those affected.

Bone marrow transplantation (BMT) is effective in preventing the progressive mental retardation in children with MPS IH (Hurler disease) if it is done before two years of age. It is less effective in correcting or preventing the bone and joint complications of the disease. BMT is considered standard treatment for infants with MPS IH if a suitable matched bone marrow donor can be found and the procedure done before the child reaches the age of two years. The principle of BMT is to replace the bone marrow, and therefore the whole blood system, of an individual affected by a particular disease with marrow from another person who is healthy. BMT is under investigation for the treatment of other lysosomal storage diseases. So far, none has shown as much benefit as patients with MPS IH (Hurler disease).

Enzyme replacement therapy (ERT) has proven effective for individuals with Gaucher disease type I. Anemia and low platelet counts have improved, enlargement of the liver and spleen have been greatly reduced, and skeletal findings have improved. These systemic manifestations also improve in individuals with Gaucher disease types II and III who receive ERT. However, ERT has not been effective in reducing or reversing neurological symptoms associated with Gaucher disease types II and III.

The orphan drug alglucerase injection (Ceredase), which is a placenta-derived enzyme, was approved by the U.S. Food and Drug Administration (FDA) in April 1991 for the treatment of Gaucher disease type I. It was the first ERT proven effective for the treatment of Gaucher disease type I, and it continues to be studied for the treatment of types II and III.

The synthetic form of this drug, imiglucerase (Cerezyme), was approved by the FDA in 1994. Recombinant DNA technology, or genetic engineering, is used to produce Cerezyme. This was an important step in overcoming limitations of the availability of Ceredase,

which is derived from human tissue sources. Ceredase and Cerezyme are manufactured by the Genzyme Corporation of Cambridge, MA. They replace, glucocerebrosidase, the enzyme that individuals with Gaucher lack.

In 2003, the FDA approved the use of miglustat (Zavesca) tablets as the first oral treatment option for individuals with Gaucher disease type I. Zavesca is the first of a new class of drugs known as substrate reduction therapy. Zavesca is used for individuals with mild to moderate Gaucher disease type I who do not respond to enzyme replacement therapy. For information on Zavesca, contact:

Actelion Pharmaceuticals Ltd.

Gewerbestrasse 16

4123 Allschwil, Switzerland

Tel: +41 61 487 45 45

Fax: +41 61 487 45 00

www.actelion.com

Enzyme replacement therapies for Fabry disease and MPS I, earlier designated as orphan drugs, were approved by the FDA in the spring of 2003.

The FDA approved Fabrazyme for Fabry disease making it the first specific treatment approved for that disease. Fabry results from a deficiency of the enzyme alpha-galactosidase A, and Fabrazyme is a version of the human form of this natural enzyme produced by recombinant DNA technology. It is given intravenously. The replacement of this missing enzyme reduces lipid accumulation in many types of cells, including blood vessels in the kidney and other organs. Fabrazyme was approved under an accelerated or early approval mechanism that expedites the approval of therapies that treat serious or life-threatening illnesses when studies indicate early favorable outcomes that are likely to predict clinical benefit. Fabrazyme is manufactured by the Genzyme Corporation of Cambridge, MA.

The FDA also granted marketing approval for Aldurazyme, the first specific treatment for MPS I, during the spring of 2003. Aldurazyme is indicated for patients with the Hurler and Hurler-Scheie forms of MPS I, and for patients with the Scheie form who have moderate to severe symptoms. The risks and benefits of treating mildly affected patients with the Scheie form have not been established.

MPS I is caused by a deficiency of the enzyme alpha L-iduronidase, leading to the accumulation of a carbohydrate called glycosaminoglycan (GAG) in tissues and organ systems.

Hurler is the most severe form of MPS I, Scheie is a milder form, and Hurler-Scheie is an intermediate form. Until the approval of Aldurazyme, it was possible to treat only the neurological symptoms of MPS I by bone marrow transplantation. Treatment with Aldurazyme is the first specific treatment for the non-neurological complications of this disease.

Enzyme replacement therapies have also been approved by FDA for Pompe disease and Hunter syndrome.

Aside from these therapies for specific disorders, much of the treatment that is currently available for lysosomal storage diseases involves treating symptoms rather than treating the underlying disease.

Investigational Therapies

Information on current clinical trials is posted on the Internet at www.clinicaltrials.gov. All studies receiving U.S. government funding, and some supported by private industry, are posted on this government web site.

For information about clinical trials being conducted at the NIH Clinical Center in Bethesda, MD, contact the NIH Patient Recruitment Office:

Tollfree: (800) 411-1222

TTY: (866) 411-1010

Email:

For information about clinical trials sponsored by private sources, contact:

www.centerwatch.com

Enzyme replacement therapies are being studied for some lysosomal storage diseases.

Gene therapy is also being studied as another possible approach to therapy for some lysosomal storage disorders.

In gene therapy, the defective gene present in a patient is replaced with a normal gene to enable the production of active enzyme and prevent pathology. Given the permanent transfer of the normal gene, which is able to produce active enzyme at all sites of disease,

this form of therapy is theoretically most likely to lead to a “cure”. However, at this time, there are many technical difficulties to resolve before gene therapy can succeed.

Resources

NORD provides referrals to many patient organizations dealing with specific lysosomal storage diseases. Please see our reports on the specific topics for those organizations. The following organizations offer general information on lysosomal storage diseases.

NORD Member Organizations

MLD Foundation
21345 Miles Drive
West Linn, OR 97068
Email:
Website: http://www.mldfoundation.org/

Other Organizations

- Genetic and Rare Diseases (GARD) Information Center
  PO Box 8126
  Gaithersburg, MD 20898-8126
  Phone: (301) 251-4925
  Toll-free: (888) 205-2311
  Website: http://rarediseases.info.nih.gov/GARD/
- Hide & Seek Foundation for Lysosomal Disease Research
  6475 East Pacific Coast Highway Suite 466
  Long Beach, CA 90803
  Phone: (877) 621-1122
  Email:
  Website: http://www.hideandseek.org
- Instituto de Errores Innatos del Metabolismo
  Pontificia Universidad Javeriana Ed. Jesús Emilio
  Ramírez (53) Laboratorios 305A - 303
  Bogota, Colombia
  Phone: (571) 320-8320
  Email:
  Website: http://www.javeriana.edu.co/ieim/programas_ieim.htm

- Lysosomal Diseases New Zealand
  167 Hollister Lane
  Ohauiti
  Tauranga, 6008 New Zealand
  Phone: 75448868
  Email:
  Website: http://www.ldnz.org.nz
- March of Dimes
  1550 Crystal Dr, Suite 1300
  Arlington, VA 22202 USA
  Phone: (888) 663-4637
  Website: http://www.marchofdimes.org
- Metabolic Support UK
  5 Hilliards Court, Sandpiper Way
  Chester Business Park
  Chester, CH4 9QP United Kingdom
  Phone: 0124420758108452412173
  Email:
  Website: https://www.metabolicsupportuk.org/
- NIH/National Institute of Child Health and Human Development
  31 Center Dr
  Building 31, Room 2A32
  Bethesda, MD 20892
  Toll-free: (800) 370-2943
  Email:
  Website: http://www.nichd.nih.gov/
- NIH/National Institute of Diabetes, Digestive & Kidney Diseases
  Office of Communications & Public Liaison
  Bldg 31, Rm 9A06
  Bethesda, MD 20892-2560
  Phone: (301) 496-3583
  Email:
  Website: http://www2.niddk.nih.gov/
- NIH/National Institute of Neurological Disorders and Stroke
  P.O. Box 5801
  Bethesda, MD 20824
  Phone: (301) 496-5751
  Toll-free: (800) 352-9424
  Website: http://www.ninds.nih.gov/

- The Arc
  1825 K Street NW, Suite 1200
  Washington, DC 20006
  Phone: (202) 534-3700
  Toll-free: (800) 433-5255
  Email:
  Website: http://www.thearc.org
- Vaincre Les Maladies Lysosomales
  2 Ter Avenue
  Massy, 91300 France
  Phone: 169754030
  Email:
  Website: http://www.vml-asso.org

References

TEXTBOOKS

Scriver CR, Beaudet AL, Sly WS, et al., eds. The Metabolic Molecular Basis of Inherited Disease. 7th ed. McGraw-Hill Companies. New York, NY; 1995:2510-2868.

Lyon G, Adams RD, Kolodny EH. Eds. Neurology of Hereditary Metabolic Diseases in Childhood. 2nd ed. McGraw-Hill Companies. New York, NY; 1996.

Alpha-N-acetylgalactosaminidase deficiency: p. 138.

GM2-Gangliosidosis: (Tay-Sachs) pp. 49-52; (Sandhoff) pp.235-37.

Glucose Storage Disease II: pp. 64-66.

Glycoprotein Disorders: pp. 162-63, 165-71.

Mucopolysaccharidoses: pp. 150-61.

Niemann-Pick Disease: pp. 55-57, 238-40.

REVIEW ARTICLES

Cox TM. Substrate reduction therapy for lysosomal storage diseases. Acta Paediatr Suppl. 2005;94:69-75.

Weinreb NJ, Barranger JA, Charrow J, et al., Guidance on the use of miglustat for treating patients with type 1 Gaucher disease. Am J Hematol. 2005;80:223-9.

Butters TD, Dwek RA, Platt FM. New therapeutics for the treatment of glycosphingolipid lysosomal storage diseases. Adv Exp Med Biol. 2003;535:219-26.

Peters C, Shapiro EG, Anderson J, Henslee PJ, Klemperer MR, Cowan MJ, et al. Hurler syndrome II. Outcome of HLA-genotypically identical sibling and HLA-haploidentical related donor bone marrow transplantation in fifty-four children. The Storage Disease Collaborative Study Group. Blood 1998;91:2601-08.

Desnick RJ, Brady R, Barranger J, et al. Fabry disease, an under-recognized multisystemic disorder: expert recommendations for diagnosis, management, and enzyme replacement therapy. Ann Intern Med. 2003;138:338-46.

Pastores GM, Barnett NL. Substrate reduction therapy: miglustat as a remedy for symptomatic patients with Gaucher disease type 1. Expert Opin Investig Drugs. 2003;12:273-81.

Bahr BA, Bendiske J. The neuropathogenic contributions of lysosomal dysfunction. J Neurochem. 2002;83:481-89.

Jeyakumar M, Butters TD, Dwek RA, et al. Glycosphingolipid lysosomal storage diseases: therapy and pathogenesis. Neuropathol Appl Neurobiol. 2002;28:343-57.

Weimer JM, Kriscenski-Perry E, Elshatory Y, et al. The neuronal ceroid lipofuscinoses: mutations in different proteins result in similar disease. Neuromolecular Med. 2002;1:111-24.

Weibel TD, Brady RO. Systematic approach to the diagnosis of lysosomal storage disorders. Mental Retard Dev Disabil Res Rev. 2001;7:190-99.

JOURNAL ARTICLES

de Grey AD. Bioremediation meets biomedicine: therapeutic translation of microbial catabolism to the lysosome. Trends Biotechnol. 2002;20:452-55.

Lacoste-Collin L, Garcia V, Uro-Coste E, et al. Danon's disease (X-linked vacuolar cardiomyopathy and myopathy): a case with a novel Lamp-2 gene mutation. Neuromuscul Disord. 2002;12:882-85.

Sugie K, Yamamoto A, Murayama K, et al. Clinicopathological features of genetically confirmed Danon disease. Neurology. 2002;58:1773-78.

Whitfield PD, Nelson P, sharp PC, et al. Correlation among genotype, phenotype, and biochemical markers in Gaucher disease: implications for the prediction of disease severity. Mol Genet Metab. 2002;75:46-5.

Winchester B. Are there useful biochemical markers of disease activity in lysosomal storage diseases? J Inherit Metab Dis. 2001;24 Suppl 2:52-56, 45-46.

Wisniewski KE, Kida E, Walus M, et al. Tripeptidyl-peptidase I in neuronal lipofuscinoses and other lysosomal diseases. Eur J paediatr Neurol. 2001;5 Suppl A:73-79.

FROM THE INTERNET

About Lysosomal Storage Diseases. Go to: http://www.ldnz.org.nz/page2.html.

LSDs At A Glance: Overview. Go to http://www.lsdn.com/glance_overview.htm

Years Published

2003, 2006

The information in NORD's Rare Disease Database is for educational purposes only and is not intended to replace the advice of a physician or other qualified medical professional.

The content of the website and databases of the National Organization for Rare Disorders (NORD) is copyrighted and may not be reproduced, copied, downloaded or disseminated, in any way, for any commercial or public purpose, without prior written authorization and approval from NORD. Individuals may print one hard copy of an individual disease for personal use, provided that content is unmodified and includes NORD's copyright.

National Organization for Rare Disorders (NORD)
55 Kenosia Ave., Danbury CT 06810 • (203)744-0100
