# Supplementary material for: Rare disease education in medical schools: patient-centered and innovative strategies
Source: Orphanet J Rare Dis. 2025 Nov 20;20:596. doi: 10.1186/s13023-025-03771-8 (PMC12632075; doi:10.1186/s13023-025-03771-8)
Supplement: Supplementary file 3 — Additional file 3. [file 13023_2025_3771_MOESM3_ESM.pdf]

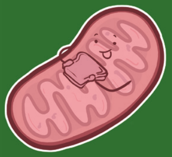

NOTES
GLYCOGEN STORAGE DISEASES (GSD)

GENERALLY, WHAT ARE THEY?

PATHOLOGY & CAUSES

- Metabolism disorders → pathological intracellular accumulation of glycogen/ metabolic products
- AKA dextrinoses/glycogenoses
- Impaired glycogen metabolism → cells without energy between meals → cell dysfunction, death
- Tissues most dependent on carbohydrates most affected
  - Liver, heart muscle, brain, skeletal muscle

CAUSES

- Enzyme deficiency in glycogen synthesis/ breakdown
- Genetic, environmental factors

COMPLICATIONS

- Rhabdomyolysis; cardiomyopathy; atrioventricular block (AV) block; renal failure (myoglobin from dead muscle cells impairs renal function); lactic acidosis due to energy being generated from triglycerides, protein; hyperuricemia, gout (lactic, uric acid excreted via same renal transport mechanism)

SIGNS & SYMPTOMS

- Symptomatic at birth/early adulthood
- Hypoglycemia, hyperlipidemia (compensatory mechanism for hypoglycemia), fatigue, hypotonia (floppy baby), hepato/splenomegaly, hypoglycemia → seizures, metabolic acidosis → hyperventilation, vomiting

DIAGNOSIS

LAB RESULTS

- Cell count, hormones, metabolites

Muscle/liver biopsy

- With periodic acid-Schiff stain
- Detects glycogen

OTHER DIAGNOSTICS

- Genetic testing

Fasting test for clinical orientation

- Hypoglycemia

TREATMENT

OTHER INTERVENTIONS

- Corrective diet
- Symptomatic therapy (cell growth factors)
- Enzyme replacement therapy for symptom relief

OSMOSIS.org 2023 Edition

GLYCOGEN STORAGE DISEASE TYPE I (GSD I)

osms.it/GSD-I

PATHOLOGY & CAUSES

- AKA von Gierke disease
- Pathological intracellular accumulation of glucose-6-phosphate (G6P) due to impaired glycogenolysis, gluconeogenesis
- *Final product of both mechanisms:* G6P
- Most common glycogen storage disease

TYPES

GSD Ia
- Mutation of *G6PC* gene

GSD Ib
- Mutation of *G6PT1* gene

GSD Ic
- Mutation of *SLC17A3* gene

CAUSES

- G6P deficiency → G6P trapped inside cell (too polar to pass through cell membrane) → severe hypoglycemia
- Autosomal recessive mutations

COMPLICATIONS

- Hepatomegaly; kidney enlargement; atherosclerosis due to hyperlipidemia; growth, development disorders; gout, kidney damage due to hyperuricemia

SIGNS & SYMPTOMS

Fasting hypoglycemia
- Blood glucose level regulation impaired (esp. between meals) → low insulin → high cortisol, glucagon

Metabolic acidosis
- Lactate cannot convert into pyruvate due to accumulation of G6P (lactate + pyruvate → G6P via gluconeogenesis) → compensatory hyperventilation, vomiting

Hypertriglyceridemia
- Compensate for chronically low insulin

Hyperuricemia
- Excess G6P in pentose phosphate pathway → high blood concentrations of uric acid → competes with other organic acids (e.g. lactic acid) for excretion in kidneys

DIAGNOSIS

DIAGNOSTIC IMAGING

Ultrasound
- Enlarged liver, kidneys

LAB RESULTS

Liver biopsy with periodic acid-Schiff stain
- Glycogen detection

OTHER DIAGNOSTICS

- Genetic testing

Fasting test
- Hypoglycemia, doesn’t respond to glucagon injection

TREATMENT

OTHER INTERVENTIONS

- Diet consisting of frequent meals high in carbohydrates
- Regulation of metabolic complications

OSMOSIS.org 2023 Edition

GLYCOGEN STORAGE DISEASE TYPE II (GSD II)

osms.it/GSD-II

PATHOLOGY & CAUSES

- AKA Pompe disease
- Pathological accumulation of glycogen in lysosome
- Mainly affects skeletal muscles, heart; also liver, nervous system

TYPES

Early/infantile onset

- Few months after birth, typically 4–8; progression much faster

Late onset

- > two years; as late as 50–60

CAUSES

- Autosomal recessive disease caused by mutation on chromosome 17
- Acid alpha-glucosidase deficiency → glycogen cannot break down, accumulates in lysosome → lysosome bursts → glycogen, lysosomal enzymes leak into cytoplasm → cell dysfunction, death

COMPLICATIONS

- Rhabdomyolysis, completely impaired motor function, respiratory/heart failure, development disorders

SIGNS & SYMPTOMS

- Progressive muscle weakness, respiratory insufficiency due to weakened diaphragm function, arrhythmia, cardiomegaly, cardiomyopathy, hepatomegaly, hypotonia, recurrent chest infections

DIAGNOSIS

DIAGNOSTIC IMAGING

X-ray

- Cardiomegaly

LAB RESULTS

- ↑ creatine kinase, lactic dehydrogenase, alanine transaminase, aspartate transaminase

Muscle biopsy

- Glycogen detection

OTHER DIAGNOSTICS

ECG

- Arrhythmia, cardiomyopathy

TREATMENT

OTHER INTERVENTIONS

- Enzyme replacement therapy

OSMOSIS.org 2023 Edition

GLYCOGEN STORAGE DISEASE TYPE III (GSD III)

osms.it/GSD-III

PATHOLOGY & CAUSES

- AKA Cori's disease, Forbes disease, limit dextrinosis
- Intracellular pathological accumulation of incompletely broken down glycogen → buildup of dextrins (intermediate products of glycogen breakdown) → osmotic pressure of cell increases → pulls water in, damages cell
- Affects liver, skeletal muscles, heart

TYPES

GSD IIIa
- Liver, muscles

GSD IIIb
- Liver

GSD IIIc
- Liver, muscles

CAUSES
- Autosomal recessive disease
- Deficiency of glycogen debranching enzyme (GDE): amylo-alpha-1,6-glucosidase, 4-alpha-glucanotransferase

COMPLICATIONS
- Hepatosplenomegaly, cardiomegaly, developmental disorders, liver failure

SIGNS & SYMPTOMS

- Hypoglycemia, hypotonia, arrhythmia, cardiomyopathy, rhabdomyolysis, muscle-related symptoms (later in life), may resemble GSD I

DIAGNOSIS

DIAGNOSTIC IMAGING

Ultrasound
- Hepatosplenomegaly

LAB RESULTS

Liver/muscle biopsy with periodic acid-Schiff stain
- Glycogen detection

OTHER DIAGNOSTICS
- Genetic analysis

TREATMENT

OTHER INTERVENTIONS
- High glucose, protein diet

OSMOSIS.org 2023 Edition

GLYCOGEN STORAGE DISEASE TYPE IV (GSD IV)

osms.it/GSD-IV

PATHOLOGY & CAUSES

- AKA Andersen's disease
- Intracellular accumulation of abnormally formed glycogen
- Improper glycogen synthesis → buildup of polyglucosan bodies (unbranched long chains of glucose) → precipitation of polyglucosan bodies → foreign body reactions, increased osmotic pressure → cell damage

CAUSES

- Autosomal recessive mutation of *GBE1* gene on chromosome 3
- Deficiency of 1,4-alpha-glucan branching enzyme (GBE)

SIGNS & SYMPTOMS

- Fatal perinatal neuromuscular
  - Fetal hydrops, hydramnion; hypotonia; reduced infant mobility; cardiomyopathy present at birth; death approx. one month after birth
- Childhood neuromuscular
  - Variable intensity, life expectancy; manifests in childhood; myopathy, cardiomegaly
- Progressive hepatic
  - Failure to thrive (FTT); liver cirrhosis; portal hypertension; ascites; death few months after birth
- Non-progressive hepatic
  - Similar to progressive hepatic type, symptoms less intense; cirrhosis not present; can live into adulthood
- Congenital muscular
  - Manifests shortly after birth; cardiomegaly; life expectancy few months

DIAGNOSIS

DIAGNOSTIC IMAGING

Prenatal ultrasound

- Fetal hydrops, hydramnion

ECG

- Heart abnormalities

LAB RESULTS

Liver/muscle biopsy with periodic acid-Schiff stain

- Glycogen detection

OTHER DIAGNOSTICS

- Genetic analysis

Echocardiography

- Heart abnormalities

TREATMENT

OTHER INTERVENTIONS

- Only symptomatic treatment available

OSMOSIS.org 2023 Edition

GLYCOGEN STORAGE DISEASE TYPE V (GSD V)

osms.it/GSD-V

PATHOLOGY & CAUSES

- AKA McArdle's disease
- Intracellular pathological accumulation of glycogen in muscle tissue
- Cannot release glucose-1-phosphate → ↓ glycolysis, cell energy status, Na^+/K^+ ATPase function → osmotic cell imbalance → cell damage
- Affects only muscle tissue

TYPES

Early onset
- More severe symptoms, progression

Adult onset
- Less severe symptoms, progression

CAUSES

- Autosomal recessive disease
- Deficiency of myophosphorylase (muscle-specific isoform of glycogen phosphorylase)

COMPLICATIONS

- Renal failure due to myoglobinuria from rhabdomyolysis
- Muscle contractures due to fibrosis caused by extensive damage

SIGNS & SYMPTOMS

- Exercise intolerance, fatigue, rhabdomyolysis, muscle fibrosis
- *"Second wind" phenomenon:* shift in metabolism during exercise → sudden burst of energy, despite being out of breath

DIAGNOSIS

LAB RESULTS

Muscle biopsy
- With periodic acid-Schiff stain
- Glycogen detection

OTHER DIAGNOSTICS

- Genetic analysis

TREATMENT

OTHER INTERVENTIONS

- Only symptomatic treatment available

OSMOSIS.org 2023 Edition

GLYCOGEN STORAGE DISEASES OVERVIEW

	ALSO KNOWN AS	DEFICIENT ENZYME	PRIMARILY AFFECTS
GSD I	von Gierke disease	glucose-6-phosphatase	Liver, kidneys
GSD II	Pompe disease	Acid alpha-glucosidase	Skeletal muscle, heart
GSD III	Cori's disease	Glycogen debranching enzyme	Liver, skeletal muscle, heart
GSD IV	Andersen's disease	Glycogen branching enzyme	Liver, skeletal muscle, heart
GSD V	McArdle's disease	Myophosphorylase	Skeletal muscle

OSMOSIS.org 2023 Edition
