# Supplementary material for: Rare disease education in medical schools: patient-centered and innovative strategies
Source: Orphanet J Rare Dis. 2025 Nov 20;20:596. doi: 10.1186/s13023-025-03771-8 (PMC12632075; doi:10.1186/s13023-025-03771-8)
Supplement: Supplementary file 4 — Additional file 4. [file 13023_2025_3771_MOESM4_ESM.pdf]

Default Question Block

Pre-Panel Survey

This survey will take you less than 5 minutes to finish.

You will receive one point towards your Biochemistry Course for submitting the pre-survey and a second point for submitting the post-survey. If you do not wish to participate in the study, please submit the surveys with the questions blank.

Please answer as many questions as HONESTLY as you can based on your CURRENT knowledge.

By taking this survey, you hereby demonstrate your acknowledgment of having read, understood the contents of this

Informed Consent Document (linked below), and express your voluntary consent to partake in this study.

Informed consent form

To link your pre and post-survey data, please create an unique multi-digit identifier using the following:

1. First letter of your mother's first name
2. Last letter of your last name
3. The DAY of your birth (please enter only the 2 digits DAY. For example: September 05, please enter "05")
4. First letter of your street address

Example: mk19t

Please REMEMBER your unique identifier to link your responses on the post-panel survey.

Has anyone in your immediate OR extended family been diagnosed with a rare disease?

- ○ Yes
- ○ No
- ○ Unsure

Have you ever been diagnosed with a rare disease?

- ○ Yes
- ○ No
- ○ Maybe
- ○ Prefer not to answer

BEFORE attending medical school, have you ever had any classes about rare diseases during your studies?

- ○ Yes
- ○ No

Have you participated in research on rare diseases?

- ○ Yes
- ○ No

Do you have volunteer experience with patients with rare diseases?

- ○ Yes
- ○ No

BEFORE medical school, have you ever searched for information on rare diseases?

- ○ Yes
- ○ No

Where did you go FIRST to get information about rare diseases?

- ○ Mandatory courses
- ○ Elective courses
- ○ Scientific literature and research
- ○ Scientific conferences and Symposia
- ○ Internet
- ○ [ ] Other

Where would you go FIRST to get information about rare diseases?

- ○ Mandatory courses
- ○ Elective courses
- ○ Scientific literature and research
- ○ Scientific conferences and Symposia
- ○ Internet

○ [ ] Other

Currently, what is the specialty you are most interested in?

- ○ Internal Medicine
- ○ Pediatrics
- ○ Obstetrics and Gynecology
- ○ Family Medicine
- ○ Emergency Medicine
- ○ General Surgery
- ○ Anesthesiology
- ○ Neurology
- ○ Psychiatry
- ○ Pathology
- ○ Combined Internal medicine pediatric
- ○ Dermatology
- ○ Otolaryngology
- ○ Neurological Surgery
- ○ Orthopedic Surgery

- ◯ Radiology
- ◯ Direct Entry Medical Genetics
- ◯ Plastic Surgery
- ◯ Urology
- ◯ Cardiothoracic Surgery
- ◯ Medical Microbiology
- ◯ Nuclear Medicine
- ◯ Ophthalmology
- ◯ Physiatry
- ◯ Public Health
- ◯ Radiation Oncology
- ◯ Vascular Surgery
- ◯ Unsure

Which doctors do you believe should be educated about rare diseases AND trained to provide care for rare disease patients? Select all that apply.

- ☐ Internist
- ☐ Family physician

- [ ] Pediatrician
- [ ] Neurologist
- [ ] Geneticist
- [ ] Psychiatrist
- [ ] Immunologist
- [ ] Neonatologist
- [ ] Oncologist
- [ ] Gynecologist
- [ ] ________ Other
- [ ] Every physician regardless of specialization

Do you consider rare diseases a major public health problem?

- ○ Yes
- ○ No
- ○ Unsure

How would you rate your CURRENT understanding of the following

aspects of rare disease care?

	I feel educated on this and I am able to explain to others this aspect of rare disease care	I feel educated on this but do not feel comfortable explaining to others this aspect of rare disease care	I do not feel educated on this and do not feel comfortable explaining this aspect of rare disease care
Challenges faced by rare disease patients in their day-to-day lives.	○	○	○
Challenges faced by rare disease patients during medical visits.	○	○	○
Challenges faced by family and caregivers of rare disease patients.	○	○	○
Resources to provide rare disease patients, their families, and/or their caregivers.	○	○	○

How would you rate your CURRENT confidence level in caring for patients with rare diseases?

○ Very confident

- Somewhat confident
- Somewhat not confident
- Very unconfident
- Uncertain

What do you think is the prevalence of all rare diseases combined?

- 1 in 2
- 1 in 5
- 1 in 10
- 1 in 100
- 1 in 1,000
- 1 in 10,000
- 1 in 100,000

What do you think is the estimated number of rare diseases?

- 100-500
- 1,000-2,000

- ○ 3,000-5,000
- ○ 6,000-8,000
- ○ 9,000-1,000
- ○ Over 10,000
- ○ I do not know

What do you think is the average time to obtain an accurate diagnosis of a rare disease?

- ○ What do you think is the average time to obtain an accurate diagnosis of a rare disease?
- ○ <1 year
- ○ 2-3 years
- ○ 4-5 years
- ○ 6-7 years
- ○ 8-9 years
- ○ 10+ years

At what age group are rare diseases most frequently diagnosed?

- Newborns
- Children
- Adolescents
- Adult
- They are present in all age groups equally
- I do not know

Please SUBMIT your responses to this survey. And then, please enter your information in this Google Form to earn your point.

Pre-panel survey confirmation google form: https://forms.gle/MDVtb3yj91qSiVch6

Powered by Qualtrics
