# Supplementary material for: Rare disease education in medical schools: patient-centered and innovative strategies
Source: Orphanet J Rare Dis. 2025 Nov 20;20:596. doi: 10.1186/s13023-025-03771-8 (PMC12632075; doi:10.1186/s13023-025-03771-8)
Supplement: Supplementary file 5 — Additional file 5. [file 13023_2025_3771_MOESM5_ESM.pdf]

Default Question Block

Post- Panel Survey This survey will take you less than 5 minutes to finish.

You will receive one point towards your Biochemistry Course for submitting the pre-survey and a second point for submitting the post-survey. If you do not wish to participate in the study, please submit the surveys with the questions blank.

Please answer as many questions as HONESTLY as you can based on your CURRENT knowledge.

By taking this survey, you hereby demonstrate your acknowledgment of having read, understood the contents of this Informed Consent Document (linked below), and express your voluntary consent to partake in this study.

Informed consent form

To link your pre and post-survey data, please RE-ENTER the unique multi-digit identifier that you used on the pre-panel survey using the following:

1. First letter of your mother's first name
2. Last letter of your last name
3. The DAY of your birth (please enter only the 2 digits DAY, for example: September 05, please enter "05")
4. First letter of you street address

Example: mk19t

Do you consider rare diseases a major public health problem?

- ○ Yes
- ○ No
- ○ Unsure

How would you rate your CURRENT understanding of the following aspects of rare disease care?

	I feel educated on this and I am able to explain to others this aspect of rare disease care	I feel educated on this but do not feel comfortable explaining to others this aspect of rare disease care	I do not feel educated on this and do not feel comfortable explaining this aspect of rare disease care
Challenges faced by rare disease patients in their day-to-day lives.	○	○	○
Challenges faced by rare disease patients during medical visits.	○	○	○
Challenges faced by family and caregivers of rare disease patients.	○	○	○

	I feel educated on this and I am able to explain to others this aspect of rare disease care	I feel educated on this but do not feel comfortable explaining to others this aspect of rare disease care	I do not feel educated on this and do not feel comfortable explaining this aspect of rare disease care
Resources to provide rare disease patients, their families, and/or their caregivers.	○	○	○

How would you rate your CURRENT confidence level in caring for patients with rare diseases?

- ○ Very confident
- ○ Somewhat confident
- ○ Somewhat not confident
- ○ Very unconfident
- ○ Uncertain

What do you think is the prevalence of all rare diseases combined?

- 1 in 2
- 1 in 5
- 1 in 10
- 1 in 100
- 1 in 1,000
- 1 in 10,000
- 1 in 100,000

What do you think is the estimated number of rare diseases?

- 100-500
- 1,000-2,000
- 3,000-5,000
- 6,000-8,000
- 9,000-1,000
- Over 10,000
- I do not know

What do you think is the average time to obtain an accurate diagnosis of a rare disease?

- <1 year
- 2-3 years
- 4-5 years
- 6-7 years
- 8-9 years
- 10+ years

At what age group are rare diseases most frequently diagnosed?

- Newborns
- Children
- Adolescents
- Adult
- They are present in all age groups equally
- I do not know

How did the panel influence your attitude toward rare disease patients?

- ○ Made me care much less
- ○ Made me care less
- ○ Did not change
- ○ Made me care more
- ○ Made me care much more

How informative did you find the panel session?

- ○ Not at all helpful
- ○ Not so helpful
- ○ Somewhat helpful
- ○ Very helpful
- ○ Extremely helpful

Please rate these resources on how helpful they were to you for the panel session.

	Not at all helpful	Not so helpful	Somewhat helpful	Very helpful	Extremely helpful
Reading assignment on Lysosomal Storage Diseases	○	○	○	○	○
Osmosis video on Lysosomal Storage Diseases	○	○	○	○	○
Reading assignments on Dyslipedemias	○	○	○	○	○
Physician Introduction to diagnostic journey and rare diseases	○	○	○	○	○
Patients sharing their rare disease diagnostic and treatment journey	○	○	○	○	○
Patient-student interactions during the Q&A	○	○	○	○	○

Please rate these resources on how important you think each one will be for your FUTURE CARE of patients with rare diseases.

	Not at all helpful	Not so helpful	Somewhat helpful	Very helpful	Extremely helpful
Patient’s clinical records	○	○	○	○	○
Clinical decision-support software (e.g., UpToDate)	○	○	○	○	○
NORD website	○	○	○	○	○
Medical school education about rare disease	○	○	○	○	○
Residency education about rare disease	○	○	○	○	○

Select the option below that best reflects your current opinion on the AMOUNT of rare disease content in the medical curriculum.

- ○ There should be LESS rare disease content in the medical curriculum
- ○ There should be MORE rare disease content in the medical curriculum
- ○ The amount of rare disease content in the medical curriculum is JUST ABOUT RIGHT

Select the option below that best reflects your current opinion about the DISTRIBUTION of rare diseases in medical education.

- ○ Rare disease content should be present only in pre-clinical years
- ○ Rare disease content should be present only in clinical years
- ○ Rare disease content should be present across all years
- ○ I do not want rare disease content in any year

Please SUBMIT your responses to this survey. And then, please enter your information in this Google Form to earn your point.

Post-panel survey confirmation google form: https://forms.gle/HknMurvRAC5nmHs27

Powered by Qualtrics
