# Supplementary material for: Rare disease education in medical schools: patient-centered and innovative strategies
Source: Orphanet J Rare Dis. 2025 Nov 20;20:596. doi: 10.1186/s13023-025-03771-8 (PMC12632075; doi:10.1186/s13023-025-03771-8)
Supplement: Supplementary file 6 — Additional file 6. [file 13023_2025_3771_MOESM6_ESM.pdf]

Rare Disease Panel Analysis

Eric Wan

2023-10-05

R Markdown

This is an R Markdown document. Markdown is a simple formatting syntax for authoring HTML, PDF, and MS Word documents. For more details on using R Markdown see http://rmarkdown.rstudio.com.

When you click the **Knit** button a document will be generated that includes both content as well as the output of any embedded R code chunks within the document. You can embed an R code chunk like this:

#set up DPI and wide images, packages, and load the data

```
## -- Attaching core tidyverse packages ------------------------ tidyverse 2.0.0 --
## v dplyr     1.1.3     v readr     2.1.4
## v forcats   1.0.0     v stringr   1.5.0
## v ggplot2   3.4.3     v tibble    3.2.1
## v lubridate 1.9.3     v tidyr     1.3.0
## v purrr     1.0.2
## -- Conflicts ------------------------------------------ tidyverse_conflicts() --
## x dplyr::filter() masks stats::filter()
## x dplyr::lag()    masks stats::lag()
## i Use the conflicted package (<http://conflicted.r-lib.org/>) to force all conflicts to become error
```

"What do you think is the prevalence of all rare diseases combined?"

(Q17 in the pre panel survey, Q7 in the post panel survey)

```
##    Min. 1st Qu.  Median    Mean 3rd Qu.    Max.    NA's 
##   1.000   4.000   5.000   5.036   6.000   7.000       2
```

```
##    Min. 1st Qu.  Median    Mean 3rd Qu.    Max.    NA's 
##   1.000   3.000   4.000   4.407   6.000   7.000       1
```

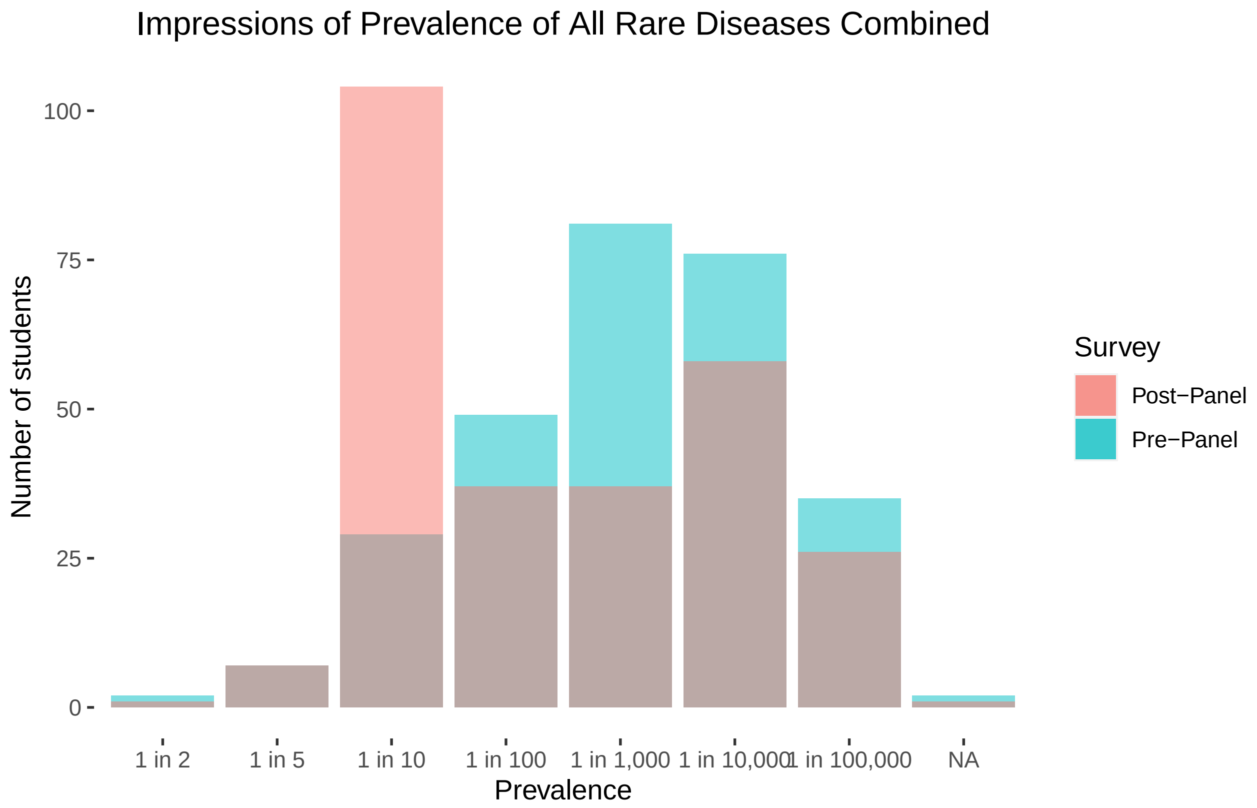


```
## 
##  Welch Two Sample t-test
## 
## data:  Pre$preQ17 and Post$postQ7
## t = 5.2402, df = 532.72, p-value = 2.315e-07
## alternative hypothesis: true difference in means is not equal to 0
## 95 percent confidence interval:
##  0.3928491 0.8640207
## sample estimates:
## mean of x mean of y 
##  5.035842  4.407407
```

“What do you think is the estimated number of rare diseases?”

(Q18 in the pre panel survey, Q8 in the post panel survey)

```
##    Min. 1st Qu.  Median    Mean 3rd Qu.    Max.    NA's 
##   1.000   3.000   4.000   4.509   6.000   7.000       2
```

```
##    Min. 1st Qu.  Median    Mean 3rd Qu.    Max.    NA's 
##   1.000   4.000   6.000   4.907   6.000   7.000       1
```

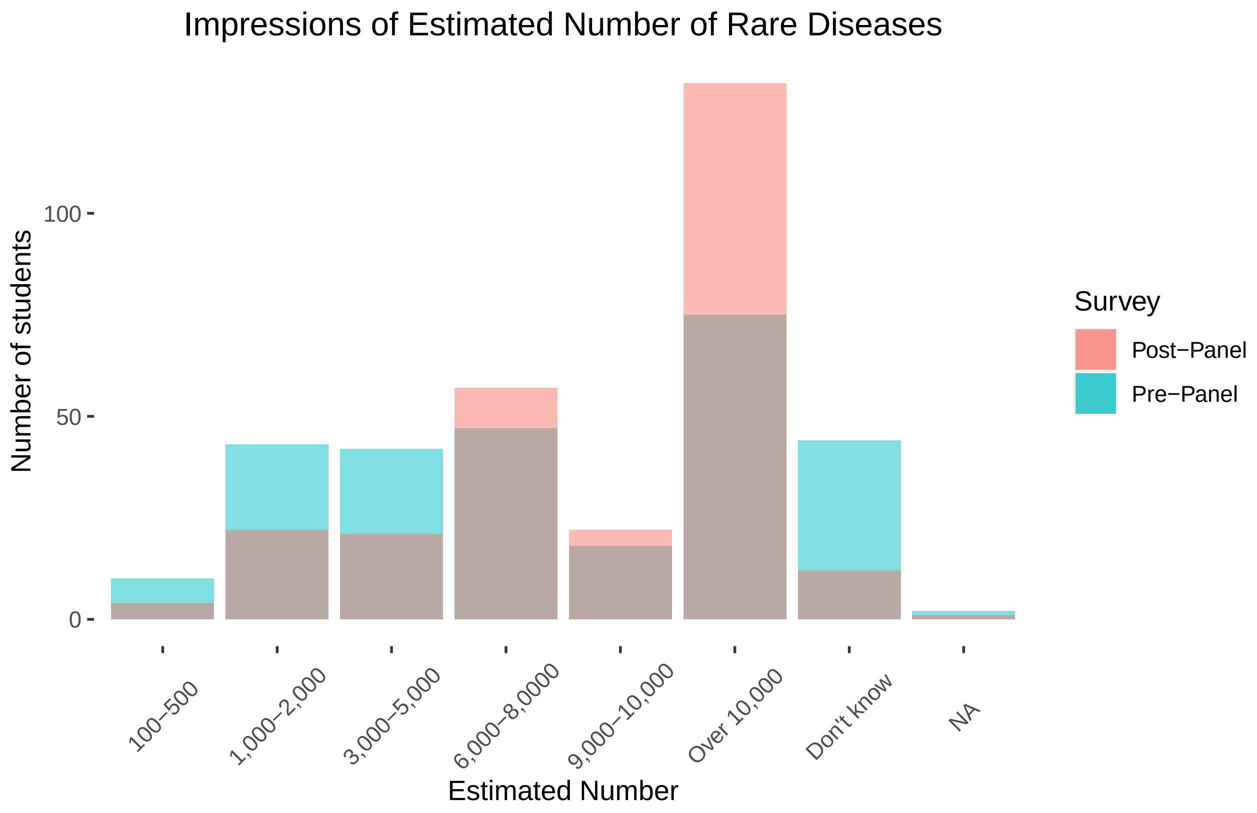


```
##              Pre Post
## 100-500       10    4
## 1,000-2,000   43   22
## 3,000-5,000   42   21
## 6,000-8,0000  47   57
## 9,000-10,000  18   22
## Over 10,000   75  132
## Don't know    44   12
```

```
## 
##  Pearson's Chi-squared test
## 
## data:  df
## X-squared = 51.565, df = 6, p-value = 2.28e-09
```

"Do you consider rare diseases a major public health problem?"

(Q14 in the pre panel survey, Q4 in the post panel survey)

```
##    Min. 1st Qu.  Median    Mean 3rd Qu.    Max.    NA's 
##   1.000   1.000   2.000   2.072   3.000   3.000       5
```

```
##    Min. 1st Qu.  Median    Mean 3rd Qu.    Max. 
##   1.000   1.000   1.000   1.173   1.000   3.000
```

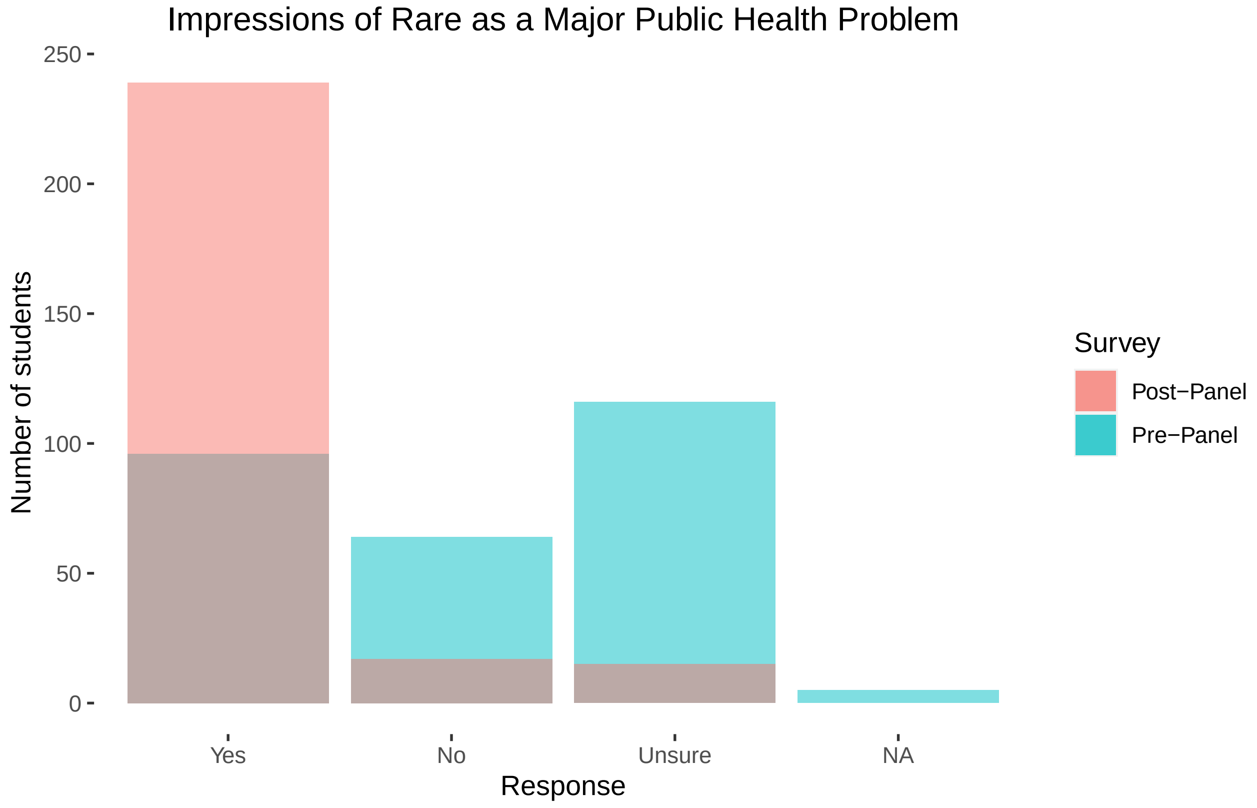


```
##        Pre Post
## Yes     96  239
## No      64   17
## Unsure 116   15
```

```
## 
##  Pearson's Chi-squared test
## 
## data:  df
## X-squared = 166.15, df = 2, p-value < 2.2e-16
```

“At what age group are rare diseases most frequently diagnosed?”

(Q20 in the pre panel survey, Q10 in the post panel survey)

```
##    Min. 1st Qu.  Median    Mean 3rd Qu.    Max.    NA's
##   1.000   3.000   4.000   3.828   5.000   6.000       2
```

```
##    Min. 1st Qu.  Median    Mean 3rd Qu.    Max.    NA's
##   1.000   1.000   2.000   2.485   3.000   6.000       1
```

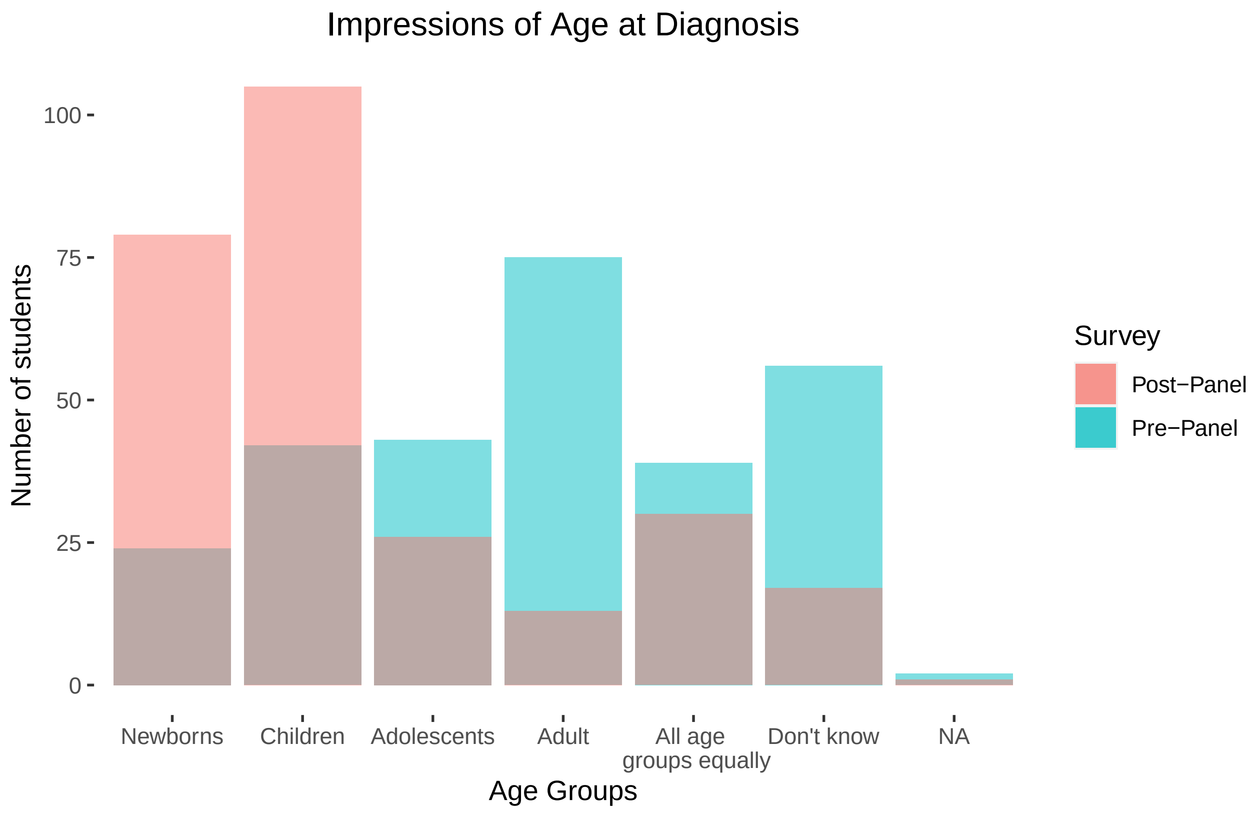


```
##                           Pre Post
## Newborns                   24   79
## Children                   42  105
## Adolescents                43   26
## Adult                      75   13
## All age \n groups equally  39   30
## Don't know                 56   17
```

```
##
##  Pearson's Chi-squared test
##
## data:  df
## X-squared = 126.14, df = 5, p-value < 2.2e-16
```

How would you rate your CURRENT confidence level in caring for patients with rare diseases?

(Q16 in the pre panel survey, Q6 in the post panel survey)

```
##    Min. 1st Qu.  Median    Mean 3rd Qu.    Max.    NA's 
##   1.000   3.500   4.000   3.796   4.000   5.000       2

##    Min. 1st Qu.  Median    Mean 3rd Qu.    Max. 
##   1.000   2.000   2.000   2.498   3.000   5.000
```

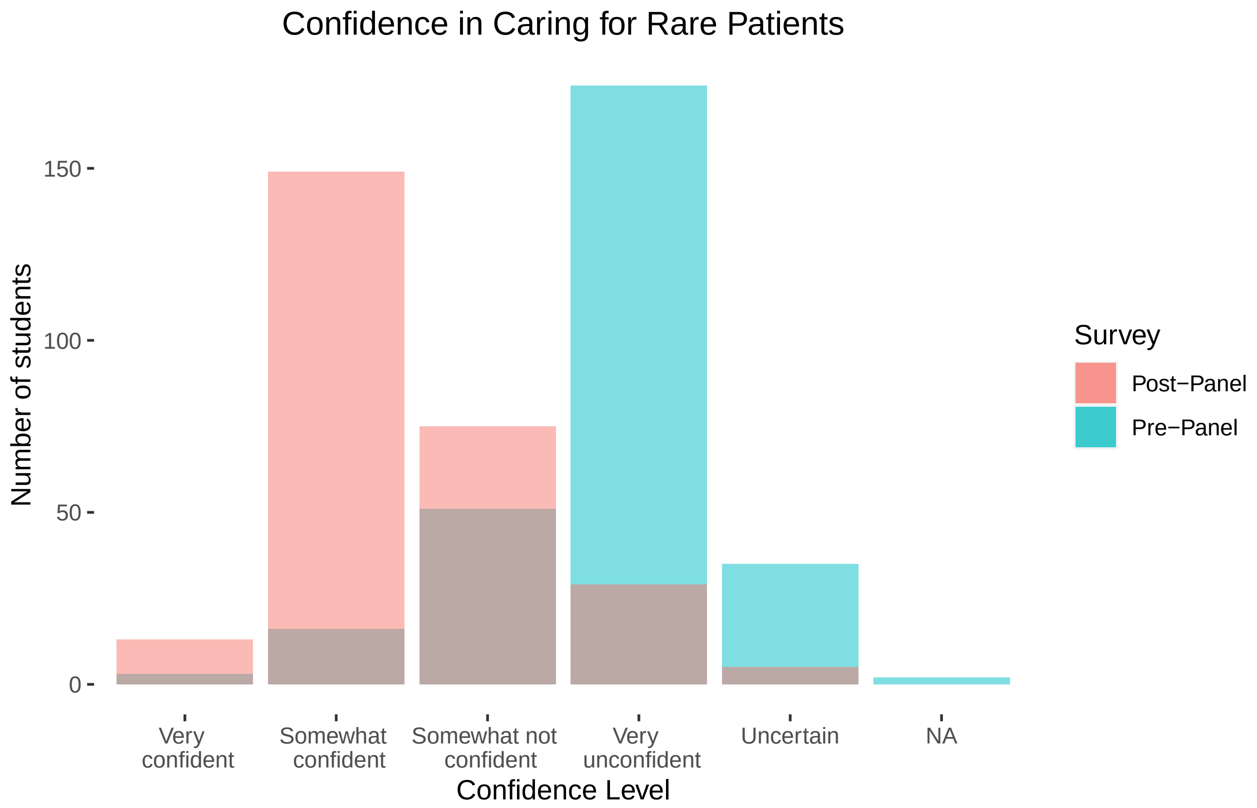


```
##                        Pre Post
## Very Confident           3   13
## Somewhat Confident      16  149
## Somewhat Not Confident  51   75
## Very Unconfident       174   29
## Uncertain               35    5

## 
##  Pearson's Chi-squared test
## 
## data:  df
## X-squared = 244.03, df = 4, p-value < 2.2e-16
```
